# Dynamic Pareto Optimization of Consolidated Bioprocessing for Ethanol Titer, Productivity, Conversion, and Operating Severity

**DOI:** 10.3390/bioengineering13060605

**Published:** 2026-05-23

**Authors:** Mark Korang Yeboah, Nana Yaw Asiedu, Ahmad Addo

**Affiliations:** 1 Chair of Dynamics and Control, University of Duisburg–Essen, Lotharstraße, 47057 Duisburg, Germany; 2 Faculty of Mechanical and Chemical Engineering, Kwame Nkrumah University of Science and Technology, Kumasi 00233, Ghana; nyasiedu.coe@knust.edu.gh (N.Y.A.); aaddo.coe@knust.edu.gh (A.A.)

**Keywords:** consolidated bioprocessing, lignocellulosic ethanol, dynamic optimization, multi-objective optimization, pareto optimality, optimal control, temperature and pH policies, bioprocess modeling

## Abstract

Consolidated bioprocessing (CBP), in which enzyme production, substrate hydrolysis, and fermentation occur in a single bioreactor, offers a promising pathway for lignocellulosic ethanol production. However, CBP operation involves competing objectives, including ethanol titer, volumetric productivity, substrate conversion, soluble sugar accumulation, batch duration, control effort, and the operating severity associated with temperature and pH profiles. This study introduces a feasibility-aware multi-objective dynamic optimization framework for identifying Pareto-optimal operating policies for batch CBP. A reduced-order mechanistic model is developed to represent biomass growth, enzyme activity, insoluble substrate hydrolysis, soluble sugar formation and consumption, ethanol production, and inhibition under time-varying temperature and pH conditions. The optimization simultaneously maximizes ethanol titer, productivity, and substrate conversion while minimizing sugar accumulation, operating severity, control movement, and batch time. In the main simulation run, 120,000 dynamic policies were retained for analysis, resulting in 5017 feasible policies and 328 feasible Pareto-optimal policies under a minimum conversion threshold of 0.42. Within the feasible Pareto archive, the highest ethanol titer reached $1.265\;g\;L^{-1}$, the highest productivity reached $0.017\;g\;L^{-1}\;h^{-1}$, and the maximum conversion reached 0.440. Compared with the best criterion-specific static constant-operation baselines, the dynamic Pareto policies improved ethanol titer, productivity, and conversion by 10.6%, 8.3%, and 14.3%, respectively. A feasibility analysis showed that a conversion threshold of 0.42 was stringent but attainable, whereas thresholds of 0.44 and 0.55 were not attainable under the present model and operating bounds. Independent-seed repetitions confirmed a consistent high-performing region across stochastic searches. The resulting Pareto fronts and operating policy maps provide a model-based decision-support basis for selecting dynamic temperature and pH profiles for CBP operation. Because this study is in silico, future experimental validation is required before direct pilot- or industrial-scale application.

## 1. Introduction

Producing renewable liquid biofuels from lignocellulosic feedstocks has received considerable research interest over the years. Various conversion strategies have been explored; however, one particularly promising approach is consolidated bioprocessing (CBP). The appeal of CBP lies in combining cellulase enzyme generation, cellulose hydrolysis, fermentation of hydrolyzed sugars, and end-product formation within a single biological process [1,2]. In principle, CBP can reduce process complexity and lower cellulase-related costs compared with conventional routes in which enzyme production, hydrolysis, and fermentation are carried out separately.

Despite these advantages, CBP remains difficult to operate and optimize because several biological and biochemical events occur simultaneously. Substrate solubilization, soluble sugar formation, microbial growth, enzyme activity, ethanol production, and inhibition effects are tightly coupled during batch operation [3,4]. This coupling creates operational conflicts because the conditions that favor one subprocess may not be optimal for another. In particular, temperature and pH influence cellulase activity, microbial growth, hydrolysis, fermentation, and ethanol productivity. Therefore, CBP operation cannot be fully described by a single performance measure, and its optimization requires simultaneous consideration of multiple competing objectives.

Prior work in bioprocess modeling has shown that dynamic models are useful for process design, scale-up, monitoring, and control [5]. In CBP, dynamic and mechanistic models have provided important insight into cellulolytic fermentation, genome-scale metabolic behavior, and the coordination of saccharification and fermentation. For example, genome-scale modeling has been used to analyze the metabolic characteristics of *Clostridium thermocellum* in relation to ethanol production [6], while coordinated enzymatic and cybernetic modeling has helped describe the coupling between cellulose hydrolysis and fermentation [7]. Kinetic studies have also examined growth, substrate utilization, product formation, and inhibition behavior in cellulolytic fermentation systems [8]. More recently, data-driven CBP modeling has broadened this perspective by showing that literature-derived CBP datasets include not only ethanol but also reported co-products with uneven support and missing-label structure [9]. Together, these studies provide valuable mechanistic, kinetic, and data-driven foundations for CBP modeling. However, they have generally not focused on constructing feasibility-aware Pareto fronts for dynamic CBP operation using time-varying temperature and pH policies.

Multi-objective optimal control provides a useful framework for analyzing systems with conflicting objectives. Unlike single-objective optimization, it produces a set of non-dominated solutions instead of one solution based on a fixed preference among objectives [10]. Multi-objective optimal experimental design has also demonstrated the value of such approaches for dynamic bioprocess systems [11]. Evolutionary Pareto search methods, including non-dominated sorting, provide practical tools for generating trade-off solutions in nonlinear optimization problems [12]. Other methods, such as normal boundary intersection and the normalized normal constraint method, have also been developed to improve the generation and interpretation of Pareto trade-off surfaces [13,14]. Although these methods are well established in general multi-objective optimization and bioprocess optimization, they have not been specifically formulated around the CBP operating conflicts among ethanol titer, productivity, substrate conversion, soluble sugar accumulation, operating severity, control movement, and batch duration.

The current study investigates the Pareto optimization of CBP operation through the construction of feasible Pareto fronts and dynamic operating policies. The manipulated variables include the time-varying temperature and pH profiles, together with the terminal batch time. The model states include biomass, enzyme activity, insoluble substrate, soluble sugar, and ethanol concentration. The process objectives involve maximizing ethanol titer, productivity, and substrate conversion while minimizing soluble sugar accumulation, temperature–pH operating severity, control movement, and batch time. Thus, the formulation is designed to identify not only high-performing policies but also the trade-offs among performance, feasibility, mildness, smoothness, and batch duration.

The novelty of the present work lies in adapting many-objective dynamic Pareto policy search specifically to the operational structure of CBP. Previous CBP studies have provided important insight into microbial cellulose utilization, cellulolytic fermentation, hydrolysis–fermentation coupling, enzyme activity, and metabolic behavior [1,2,3,6,7,8]. However, these studies have generally not developed a feasibility-aware dynamic Pareto framework for selecting time-varying CBP temperature and pH policies. Similarly, while Pareto-based dynamic optimization has been applied in broader bioprocess and multi-objective optimization contexts [10,12,13,14], these studies are not formulated around the specific CBP trade-offs considered here.

The principal contribution of this study is therefore a CBP-specific, feasibility-aware dynamic Pareto policy-search framework. This framework integrates a reduced-order CBP dynamic model, direct temperature–pH policy parameterization, feasible archive screening, static baseline comparison, conversion threshold sensitivity analysis, and independent-seed repeatability analysis. Using the resulting Pareto fronts and operating policy maps, this framework makes it possible to identify regions where ethanol productivity, substrate conversion, operating mildness, and controllability can be jointly maintained, as well as regions where strong trade-offs are unavoidable. This provides a model-based decision-support basis for selecting dynamic CBP operating policies under different process priorities. The positioning of the present study relative to published CBP modeling and Pareto-based bioprocess optimization studies is summarized in Table 1.

The rest of this paper is organized as follows. Section 2 introduces the dynamic CBP model and the state, control, and kinetic equations. Section 3 formulates the multi-objective dynamic optimization of CBP operation, including objective functions, feasibility constraints, control policies, and Pareto generation. Section 4 analyzes the resulting Pareto fronts and dynamic operating policies, including representative dynamic trajectories, Pareto policy maps, feasibility sensitivity, and repeatability. Section 5 concludes this study.

## 2. Dynamic Model of Consolidated Bioprocessing

A reduced-order dynamic model was used to represent batch CBP under time-varying temperature and pH conditions. The purpose of the model is not to describe every biological, metabolic, or structural detail of the system. In particular, it does not resolve the complete intracellular metabolic network of the microorganism, nor does it describe the distributed structure, accessibility, and composition of individual lignocellulosic particles. Instead, the model provides a compact grey-box description of the main process mechanisms that are most relevant for multi-objective dynamic optimization. These mechanisms include microbial growth, enzyme formation, cellulose hydrolysis to soluble sugars, sugar accumulation when hydrolysis is faster than fermentation, sugar consumption during ethanol production, and ethanol-related inhibition.

This level of model abstraction was selected to make large-scale policy screening computationally feasible. In the present study, many candidate temperature and pH trajectories must be simulated repeatedly in order to construct feasible Pareto fronts. A highly detailed metabolic or particle-scale model would provide greater mechanistic resolution, but it would also substantially increase the computational burden and reduce the practicality of the optimization workflow. Compact dynamic models are therefore widely used in bioprocess optimization and scale-up studies when the main goal is to compare operating strategies rather than to resolve all intracellular or microscale phenomena [5,10].

At the same time, these simplifications affect how the results should be interpreted. Treating the lignocellulosic feedstock as a lumped insoluble substrate pool neglects substrate heterogeneity, particle size effects, cellulose accessibility, and differences among the cellulose, hemicellulose, and lignin fractions. Similarly, representing enzyme action through a lumped enzyme activity variable does not distinguish among individual cellulase components or enzyme adsorption and deactivation mechanisms. The model also focuses on ethanol as the main product, while other fermentation products such as acetate, lactate, hydrogen, carbon dioxide, and organic acids are not explicitly tracked. These assumptions may influence the predicted ethanol titer, soluble sugar accumulation, inhibition behavior, and substrate conversion, especially when the model is extrapolated beyond the operating conditions used for simulation.

For this reason, the Pareto-optimal policies obtained in this study should be interpreted as model-based operating trends and decision-support results rather than as directly validated industrial-scale operating recipes. The model is intended to identify useful trade-offs among ethanol titer, productivity, conversion, sugar accumulation, operating severity, control effort, and batch time. Before direct pilot- or industrial-scale application, the predicted policies would require validation with time-resolved experimental data and, where necessary, extension of the model to include feedstock heterogeneity, co-product formation, transport limitations, imperfect mixing, heat transfer constraints, and uncertainty in kinetic parameters.

Despite these limitations, the reduced-order structure retains the key features that distinguish CBP from separate hydrolysis and fermentation processes for microbial cellulose utilization [1,2]. More detailed CBP models, including coordinated enzymatic, cybernetic, and genome-scale models, can provide deeper biological insight, but they are generally more computationally demanding for large-scale dynamic optimization [6,7,15]. The present grey-box model therefore represents a compromise between mechanistic interpretability and computational efficiency.

### 2.1. Process Description

The modeled process involves batch CBP in a well-mixed reactor, in which the microorganism converts an insoluble lignocellulosic substrate to ethanol through multiple biological and biochemical steps. First, microbial cells grow in the presence of the insoluble substrate and produce hydrolytic enzymes. Second, the enzymes promote the conversion of the insoluble substrate to soluble sugar, which is then consumed by the cells for further ethanol production. This sequence of reactions resembles the overall concept of CBP developed for microbial cellulose utilization [1]. More recent efforts have focused on developing coordinated enzymatic control strategies for cellulolytic systems such as that of *Clostridium thermocellum* [3].

Hydrolysis and fermentation occur in parallel and are essential in this model. If the hydrolysis rate exceeds the fermentation rate, the soluble sugar pool will accumulate; similarly, excessive fermentation demand can reduce the soluble sugar pool. Both temperature and pH affect the kinetics, yet the ideal conditions for each rate can be different, thus leading to an inherent operational conflict. Coordinated enzymatic modeling of *C. thermocellum* has indicated that cellulose solubilization and ethanol fermentation are tightly coupled phenomena in CBP [7]. Moreover, kinetic analysis of *C. thermocellum* fermentation has shown the significant role of growth, inhibition, and substrate-dependent kinetics [8].

While the current model is relatively compact, genome-scale and core kinetic metabolic models can provide more insight into intracellular metabolic processes [6,16]. Also, cybernetic and population balance descriptions may provide further detail on enzyme distribution in the environment or the heterogeneous structure of cellulose particles [7]. However, such a level of detail is not necessary here because the goal is to generate a simplified dynamic model for large-scale simulation and policy archive evaluation. Thus, the model should be simple enough to simulate efficiently while still providing sufficient information about the main process couplings.

### 2.2. States and Manipulated Variables

The dynamic state vector in the problem is represented as

(1) $$x\left(t\right)={\left[\begin{array}{ccccc} X(t) & E(t) & B(t) & C(t) & P(t) \end{array}\right]}^{⊤},$$

where *X*, *E*, *B*, *C*, and *P* denote biomass concentration, total enzyme activity, the insoluble substrate pool, the soluble sugar pool, and ethanol concentration, respectively. The use of biomass, substrate, sugar, and product pools follows typical bioreactor dynamic representations reported in the literature [5,8]. The state variables and manipulated variables considered in the model are summarized in Table 2.

The manipulated variable vector is

(2) $$u\left(t\right)={\left[\begin{array}{cc} T(t) & \mathrm{pH}(t) \end{array}\right]}^{⊤},$$

where T(t) and pH(t) denote the reactor temperature and reactor pH, respectively. In addition to temperature and pH, the final batch time, $t_{f}$, is considered an optimization decision. The manipulated variables were selected because of their strong influence on cellulase activity, microbial growth, and fermentation in lignocellulosic bioprocessing [3,7]. Temperature and pH were restricted to the operating ranges

(3) 30≤T(t)≤55,5.0≤pH(t)≤8.0.

These bounds define the admissible operating window used in the dynamic policy search. In the present formulation, the temperature and pH trajectories are represented as piecewise-constant profiles over the batch time. Large changes between consecutive control intervals are discouraged through the control-movement objective introduced later in the optimization formulation. However, equipment-specific hard rate-of-change constraints were not imposed in the main simulations. This choice was made because the allowable heating rate, cooling rate, and pH adjustment rate depend strongly on the reactor volume, heat transfer area, mixing efficiency, buffering capacity, and acid/base dosing system.

For practical implementation, the formulation can be extended by adding explicit ramp rate constraints between consecutive control intervals. For a policy with $N_{u}$ control intervals and an interval duration $\Delta t_{j}$, these constraints may be written as

(4) $$\left|\frac{T_{j+1}-T_{j}}{\Delta t_{j}}\right|\leq r_{T}^{\max},\;\left|\frac{{\mathrm{pH}}_{j+1}-{\mathrm{pH}}_{j}}{\Delta t_{j}}\right|\leq r_{\mathrm{pH}}^{\max},\;j=1,\ldots ,N_{u}-1,$$

where $r_{T}^{\max}$ and $r_{\mathrm{pH}}^{\max}$ denote the maximum allowable temperature and pH adjustment rates, respectively. In this study, Equation (4) is presented as a practical extension rather than as a hard constraint enforced in the main optimization. Therefore, the optimized temperature and pH profiles should be interpreted as idealized dynamic operating policies for identifying useful process trends, not as final equipment-level control recipes.

### 2.3. Phase-Weighted Kinetic Structure

The dynamic CBP model takes the form

(5) $$\frac{dx(t)}{dt}=f\left(x\left(t\right),u\left(t\right),\theta \right),\;x\left(0\right)=x_{0},$$

where θ denotes the kinetic parameter set. In this study, θ contains the nominal parameters used to describe biomass growth, enzyme formation, enzyme deactivation, substrate hydrolysis, soluble sugar consumption, ethanol formation, ethanol inhibition, and the temperature–pH activity corrections. These parameters were not obtained from new experiments in the present work and were not optimized during the Pareto search. Instead, they were selected as literature-informed nominal values to define a representative in silico CBP process suitable for dynamic policy screening. The selected values were guided by reported kinetic behavior of cellulolytic fermentation, hydrolysis–fermentation coupling, and reduced-order bioprocess modeling studies [5,6,7,8,16].

The role of the kinetic parameter set in the present study is therefore to provide a stable virtual CBP system for comparing alternative temperature and pH policies. The optimized decision variables are the temperature trajectory, the pH trajectory, and the final batch time, while the kinetic parameters are kept fixed during the optimization. Thus, the reported Pareto policies should be interpreted as model-based operating trends obtained under the assumed nominal parameter set. Future experimental work could re-estimate θ from time-resolved CBP measurements and repeat the Pareto optimization with experimentally identified parameters. Table 3 summarizes the role of each kinetic parameter group and identifies the representative literature used to guide the nominal parameter selection.

Phase-weighting functions are used to describe the transition through the growth, hydrolysis, and fermentation phases, as follows:

(6) $$\begin{array}{cc} \omega_{1}\left(t\right) & =\frac{1}{1+\exp\left[k(t-t_{1})\right]}, \end{array}$$

(7) $$\begin{array}{cc} \omega_{3}\left(t\right) & =\frac{1}{1+\exp\left[-k(t-t_{2})\right]}, \end{array}$$

(8) $$\begin{array}{cc} \omega_{2}\left(t\right) & =\max\left(0,1-\omega_{1}\left(t\right)-\omega_{3}\left(t\right)\right), \end{array}$$

where $\omega_{1}$, $\omega_{2}$, and $\omega_{3}$ denote the relative dominance of growth/enzyme formation, hydrolysis, and fermentation-related activity, respectively. These weighting functions are not intended to represent strict biological on–off switches. In batch CBP, microbial growth, enzyme formation, cellulose hydrolysis, soluble sugar consumption, and ethanol production occur simultaneously and overlap in time. However, experimental and kinetic studies of cellulolytic fermentation show that different subprocesses can dominate at different stages of the batch. Early operation is typically associated with active cell growth and enzyme formation, the intermediate period is strongly affected by cellulose solubilization and soluble sugar formation, and later operation is increasingly influenced by sugar consumption, ethanol production, and product inhibition [3,7,8]. The biological interpretation of each phase-weighting function is summarized in Table 4.

The phase-weighting functions are therefore used as a reduced-order device to represent this gradual shift in dominant process behavior while avoiding discontinuous switching between kinetic regimes. The parameters $t_{1}$, $t_{2}$, and *k* determine the approximate timing and smoothness of these transitions. In the simulations, the values $t_{1}=18$ h, $t_{2}=44$ h, and k=0.28 were selected to produce a smooth progression from early growth/enzyme formation through a hydrolysis-dominant period, and then to a later fermentation-dominant period. These values should be interpreted as nominal transition parameters for the virtual CBP system rather than experimentally identified phase boundary times. Future experimental calibration could estimate these transition parameters from time-resolved measurements of biomass, enzyme activity, residual substrate, soluble sugars, and ethanol.

Growth and enzyme dynamics are described by

(9) $$\begin{array}{cc} \frac{dX}{dt}\; & =\omega_{1}\left(t\right)\left[\left(\mu \left(T,\mathrm{pH},P\right)-k_{d}\left(P\right)\right)X\left(1-\frac{X}{K_{X}}\right)\right], \end{array}$$

(10) $$\begin{array}{cc} \frac{dE}{dt}\; & =\omega_{1}\left(t\right)\left[Y_{E}\left(T,\mathrm{pH}\right)X-k_{E}\left(T\right)E\right], \end{array}$$

where μ, $k_{d}$, $K_{X}$, $Y_{E}$, and $k_{E}$ denote the growth rate depending on temperature, pH, and ethanol concentration; the ethanol-associated deactivation coefficient; biomass carrying capacity; enzyme formation yield; and the enzyme deactivation rate, respectively. This compact growth and enzyme formation structure is used because the goal of the model is dynamic policy optimization rather than detailed intracellular metabolic reconstruction. Similar reduced-order growth descriptions are commonly used in batch fermentation and bioprocess optimization studies [8].

Hydrolysis of the insoluble substrate to soluble sugar is described as

(11) $$\begin{array}{cc} v_{h}\left(t\right)\; & =V_{h}\left(T,\mathrm{pH},S_{0}\right)\frac{B(t)}{K_{h}+B\left(t\right)}\tanh\left(E\left(t\right)\right), \end{array}$$

(12) $$\begin{array}{cc} \frac{dB}{dt}\; & =-\omega_{2}\left(t\right)v_{h}\left(t\right), \end{array}$$

where $B/(K_{h}+B)$ represents the nonlinear dependence of hydrolysis on an accessible insoluble substrate; $V_{h}$ is the maximum hydrolysis rate affected by temperature, pH, and substrate loading; and tanh(E) represents the saturating effect of enzyme-mediated catalysis. Although compact, this structure preserves the key idea that hydrolysis depends jointly on available substrate and enzyme activity. More detailed models may describe cellulase adsorption, substrate accessibility, and enzyme allocation more explicitly, but such detail would increase the computational cost of the many-policy Pareto search [7].

The soluble sugar balance is given by

(13) $$\frac{dC}{dt}=\omega_{2}\left(t\right)\left[v_{h}\left(t\right)-k_{C}C\left(t\right)\right]-\omega_{3}\left(t\right)v_{p}\left(t\right),$$

where $k_{C}$ denotes the sugar consumption coefficient during the hydrolysis-dominant phase. This balance is important because it captures one of the main features of CBP: soluble sugars may accumulate when substrate hydrolysis is faster than fermentation, but they are consumed as ethanol production becomes dominant.

Ethanol production is represented by

(14) $$\begin{array}{cc} v_{p}\left(t\right)\; & =Y_{P}\left(T,\mathrm{pH}\right)\frac{C(t)}{1+k_{I}\left(T,\mathrm{pH}\right)P\left(t\right)}, \end{array}$$

(15) $$\begin{array}{cc} \frac{dP}{dt}\; & =\omega_{3}\left(t\right)v_{p}\left(t\right), \end{array}$$

where $Y_{P}$ and $k_{I}$ denote the ethanol production coefficient and ethanol inhibition coefficient, respectively. The denominator accounts for the reduction in the effective fermentation rate as ethanol accumulates, which is consistent with inhibition terms commonly used in fermentation kinetics and CBP modeling studies [8,16].

### 2.4. Temperature- and pH-Dependent Activity Functions

The effect of temperature and pH is captured by activity functions associated with growth, hydrolysis, and fermentation. Separate activity functions are used because the most favorable conditions for microbial growth, enzyme-mediated hydrolysis, and ethanol production are not necessarily the same. Such temperature–pH conflicts have been reported in integrated hydrolysis–fermentation systems, where conditions that favor enzymatic saccharification may differ from those that favor microbial ethanol production [17]. A similar conflict arises in CBP because enzyme production, substrate hydrolysis, and fermentation occur simultaneously in the same reactor [3,7].

For each kinetic process (z=g,h,f, denoting growth, hydrolysis, and fermentation, respectively), the temperature activity function is expressed as

(16) $$\phi_{z}\left(T\right)=\mathrm{clip}\left[\exp\left(-{\left(\frac{T-T_{z}^{*}}{\sigma_{T,z}}\right)}^{2}\right),\phi_{\min},\phi_{\max}\right],$$

and the pH activity function is expressed as

(17) $$\psi_{z}\left(\mathrm{pH}\right)=\mathrm{clip}\left[\exp\left(-{\left(\frac{\mathrm{pH}-{\mathrm{pH}}_{z}^{*}}{\sigma_{\mathrm{pH},z}}\right)}^{2}\right),\psi_{\min},\psi_{\max}\right],$$

where $T_{z}^{*}$ and ${\mathrm{pH}}_{z}^{*}$ denote the nominal centers of the activity distributions, while $\sigma_{T,z}$ and $\sigma_{\mathrm{pH},z}$ control the widths of the temperature and pH response curves. The clipping operation prevents the correction factors from becoming unrealistically small or large during the policy search, which improves numerical stability during repeated dynamic simulations.

The nominal temperature and pH activity parameters used in the simulations are listed in Table 5. These values were selected to represent the expected conflict among the main CBP subprocesses. Growth and enzyme formation are centered around an intermediate condition, hydrolysis is favored at a slightly higher temperature and lower pH, and fermentation is favored at a lower temperature and slightly higher pH.

In Table 5, $T_{z}^{*}$ and $\sigma_{T,z}$ are given in °C, while ${\mathrm{pH}}_{z}^{*}$ and $\sigma_{\mathrm{pH},z}$ are expressed in pH units. The parameters $\phi_{\min}$, $\phi_{\max}$, $\psi_{\min}$, and $\psi_{\max}$ define the lower and upper clipping limits applied to the activity correction factors.

The growth, hydrolysis, and fermentation coefficients are then written as

(18) $$\begin{array}{cc} \mu (T,\mathrm{pH},P)\; & =\mu_{\max}\phi_{g}\left(T\right)\psi_{g}\left(\mathrm{pH}\right), \end{array}$$

(19) $$\begin{array}{cc} Y_{E}\left(T,\mathrm{pH}\right)\; & =Y_{E,\max}\phi_{g}\left(T\right)\psi_{g}\left(\mathrm{pH}\right)\chi_{\mathrm{pret}}, \end{array}$$

(20) $$\begin{array}{cc} V_{h}\left(T,\mathrm{pH},S_{0}\right)\; & =V_{h,\max}\phi_{h}\left(T\right)\psi_{h}\left(\mathrm{pH}\right)\chi_{\mathrm{pret}}\chi_{S}, \end{array}$$

(21) $$\begin{array}{cc} Y_{P}\left(T,\mathrm{pH}\right)\; & =Y_{P,\max}\phi_{f}\left(T\right)\psi_{f}\left(\mathrm{pH}\right), \end{array}$$

where $\chi_{\mathrm{pret}}$ and $\chi_{S}$ are correction factors representing the nominal effects of pretreatment and substrate loading, respectively. The kinetic constants and correction factors used in the main simulations are summarized in Table 6. These values were kept fixed during the Pareto search; only the temperature trajectory, pH trajectory, and final batch time were optimized.

In the main simulations, the pretreatment correction factor was set to $\chi_{\mathrm{pret}}=1.10$, representing the nominal pretreated-substrate condition, while the substrate-loading correction factor was set to $\chi_{S}=1.00$. Thus, the hydrolysis coefficient used in the model was evaluated as

(22) $$V_{h}\left(T,\mathrm{pH},S_{0}\right)=V_{h,\max}\phi_{h}\left(T\right)\psi_{h}\left(\mathrm{pH}\right)\chi_{\mathrm{pret}}\chi_{S},$$

with $V_{h,\max}=0.235\;h^{-1}$, $\chi_{\mathrm{pret}}=1.10$, and $\chi_{S}=1.00$. The apparent hydrolysis saturation constant was evaluated as

(23) $$K_{h}=\frac{0.85}{\chi_{\mathrm{pret}}\chi_{S}}.$$

The correction factors were not optimized during the Pareto search. They were kept fixed to define the nominal substrate and pretreatment condition under which the alternative temperature and pH policies were compared.

The separation between the nominal optima in Table 5 provides the main motivation for dynamic operation. Since growth, hydrolysis, and fermentation cannot all be favored simultaneously under one constant temperature and pH condition, dynamic operation allows the process to emphasize different subprocesses at different stages of the batch cycle. This is why time-varying temperature and pH policies can outperform constant operating schedules in the present CBP optimization framework [10,17].

### 2.5. Severity and Feasibility Quantities

The concept of severity is not incorporated into the state variables explicitly. Instead, the instantaneous severity function is derived from the temperature and pH dynamics and serves as an optimization objective. By using severity as an objective variable, the optimizer can differentiate policies that produce similar ethanol amounts or conversions under different severity levels. The severity penalty function depends on the temperature and pH deviations from reference values that correspond to mild operating regimes, as follows:

(24) $$s_{\mathrm{sev}}\left(t\right)={\left(\frac{T\left(t\right)-T_{\mathrm{ref}}}{\Delta T}\right)}^{2}+{\left(\frac{\mathrm{pH}\left(t\right)-{\mathrm{pH}}_{\mathrm{ref}}}{\Delta \mathrm{pH}}\right)}^{2}.$$

The severity objective is then defined as the time average of the corresponding instantaneous penalties, as follows:

(25) $$J_{\mathrm{sev}}=\frac{1}{t_{f}}\int_{0}^{t_{f}}s_{\mathrm{sev}}\left(t\right)\;dt.$$

Normalizing severity makes it comparable across policies with different batch durations. The averaging procedure is a well-established way to quantify severity in biochemical operations [15]. Penalizing extreme temperature and pH conditions is appropriate for CBP because such conditions may enhance selected kinetic processes while also increasing control complexity or reducing biological tolerance [3].

Control effort is defined in terms of changes in the piecewise-constant temperature and pH trajectories, as follows:

(26) $$J_{\Delta u}=\sum_{j=1}^{N_{u}-1}\left[{\left(T_{j+1}-T_{j}\right)}^{2}+{\left({\mathrm{pH}}_{j+1}-{\mathrm{pH}}_{j}\right)}^{2}\right].$$

Minimizing control effort is a common approach to improving operational feasibility while retaining the benefits of time-varying policies [5,10].

Conversion is calculated by comparing the insoluble substrate pool size at the start and end of the batch, as follows:

(27) $$X_{\mathrm{conv}}=\frac{B\left(0\right)-B\left(t_{f}\right)}{B(0)}.$$

A policy is said to be feasible if it meets the conversion threshold and the soluble-sugar constraint, as follows:

(28) $$X_{\mathrm{conv}}\geq X_{\mathrm{conv},\min},\;C\left(t\right)\leq C_{\max}.$$

The conversion threshold used in the main study was set to $X_{\mathrm{conv},\min}=0.42$, and the sugar concentration threshold was set to $C_{\max}=6\;g\;L^{-1}$. The value $X_{\mathrm{conv},\min}=0.42$ was selected as a stringent model-based substrate utilization requirement rather than as an arbitrary computational cutoff. In CBP, substrate conversion is a key indicator of whether the coupled hydrolysis–fermentation process is effectively using the available lignocellulosic material. A very low conversion threshold would allow policies that produce ethanol while leaving most of the insoluble substrate unused, whereas an excessively high threshold could exclude all policies within the current operating bounds and nominal kinetic parameterization. The value of 0.42 was therefore chosen to lie near the upper attainable conversion region of the reduced-order model while still retaining a non-empty feasible policy set for Pareto analysis. This makes the feasibility definition restrictive enough to exclude weak substrate utilization policies but not so restrictive that the optimization becomes infeasible.

Because the attainable conversion is model-dependent, this threshold was not treated as a universal industrial target. Instead, it was evaluated through a conversion threshold sensitivity analysis using lower and higher cutoff values. This analysis was included to determine whether the main conclusions were dependent on a single feasibility definition and to distinguish between permissive, stringent-but-attainable, and unattainable conversion requirements under the present model. A threshold for sugar accumulation was also included because a high soluble sugar concentration indicates an imbalance between hydrolysis and fermentation, which is a fundamental issue in CBP systems [7].

### 2.6. Model Assumptions

The proposed model is based on several simplifying assumptions. These assumptions were introduced to keep the model computationally efficient for large-scale dynamic policy screening, but they also define the limits within which the results should be interpreted.

First, the reactor is assumed to be perfectly mixed. Thus, biomass, enzyme activity, insoluble substrate, soluble sugar, ethanol, temperature, and pH are treated as spatially uniform throughout the reactor volume. This assumption is reasonable for a reduced-order simulation model, especially when the main aim is to compare operating policies. However, it may become less accurate at pilot or industrial scale, where mixing limitations, heat transfer gradients, mass transfer resistance, and local variations in pH or substrate concentration can influence CBP performance [5].

Second, the lignocellulosic feedstock is represented by a single lumped insoluble substrate pool, while soluble sugars are also represented as one lumped pool. This simplification preserves the main hydrolysis–fermentation coupling that is central to CBP, but it does not distinguish among cellulose, hemicellulose, lignin, oligomers, and individual monomeric sugars. It also neglects particle-size effects, substrate accessibility, crystallinity, and pretreatment-dependent structural changes. As a result, the model may not fully capture feedstock-specific hydrolysis behavior or the variability expected when different lignocellulosic materials are used.

Third, enzyme activity is represented by a single lumped activity variable. This allows the model to describe the overall contribution of enzyme formation and substrate hydrolysis without explicitly tracking individual cellulase components, enzyme adsorption, desorption, synergistic enzyme action, or enzyme deactivation pathways. More detailed cellulase kinetic and coordinated enzymatic control models can represent these effects more explicitly [7]. In the present work, however, a lumped enzyme description was preferred because it is more suitable for repeated simulation during Pareto front generation.

Fourth, ethanol is treated as the only explicit fermentation product. Other metabolites, including acetate, lactate, hydrogen, carbon dioxide, and organic acids, are not modeled as separate state variables. Their effects are instead absorbed into the effective yield and inhibition terms. This is acceptable for an ethanol-focused optimization study, but it may reduce predictive accuracy when carbon redistribution, by-product inhibition, or broader biorefinery objectives are important. Previous multi-product CBP studies have shown that co-product formation can become important when the process is evaluated beyond ethanol production alone [9].

Fifth, temperature and pH are assumed to be directly controllable within prescribed bounds and are implemented as piecewise-constant control profiles. This makes the dynamic optimization problem tractable and interpretable. In practice, however, heating and cooling rates, pH adjustment dynamics, actuator limits, sensor delays, and control system constraints may restrict how quickly these profiles can be implemented. Therefore, the optimized profiles should be viewed as idealized operating policies rather than direct plant-level control recipes.

Finally, the present formulation is deterministic. It does not explicitly account for uncertainty in kinetic parameters, feedstock composition, inoculum quality, contamination risk, measurement noise, or process disturbances. These uncertainties can be significant in CBP systems and may affect the robustness of the predicted Pareto-optimal policies. Robust or uncertainty-aware optimization would therefore be required before experimental or industrial implementation [18,19].

Overall, these assumptions make the model suitable for fast-throughput dynamic optimization and Pareto front computation. The results should therefore be interpreted as model-based decision-support trends that reveal trade-offs among ethanol titer, productivity, conversion, sugar accumulation, operating severity, control effort, and batch time. They should not be interpreted as experimentally validated kinetic optima or directly transferable industrial operating conditions. Future extensions could include feedstock heterogeneity, explicit co-product formation, transport limitations, uncertainty propagation, and validation against time-resolved experimental data. More detailed approaches, including genome-scale models, kinetic metabolic models, cybernetic models, and population-balance descriptions of cellulose depolymerization, provide useful benchmarks for such extensions [6,7,16]. However, such models would substantially increase the computational cost of the many-policy Pareto analysis performed in this study.

## 3. Formulation of the Multi-Objective Dynamic Optimization Problem

The dynamic CBP model described in Section 2 represents a nonlinear batch process in which the operating policy is the temperature and pH profile over time. The optimization task is not to find a single globally optimal policy but to generate a set of policies with different characteristics that highlight the trade-offs among ethanol titer, productivity, substrate conversion, sugar accumulation, operating severity, control movement, and batch time. Previous studies have shown that multi-objective optimal control is effective for analyzing dynamic bioprocesses because it can uncover operating alternatives that may not be found by collapsing several competing objectives into a single scalarized objective [10]. Similar results have been obtained in optimal experimental design, where dynamic biological systems often need to balance several conflicting criteria [11]. Here, a non-dominated sorting algorithm is applied to preserve non-dominated policies based on the Pareto ranking concept common to evolutionary multi-objective optimization algorithms [12]. Other scalarizing approaches, such as normal boundary intersection and the normalized normal constraint method, can serve as benchmarks for finding Pareto fronts [13,14]; however, a direct policy-search formulation is used in this paper because it allows efficient exploration of a large archive of nonlinear dynamic CBP policies without solving separate nonlinear programming problems for each preference vector.

### 3.1. Optimal Control Problem

The dynamic state vector is written as

(29) $$x\left(t\right)={\left[\begin{array}{ccccc} X(t) & E(t) & B(t) & C(t) & P(t) \end{array}\right]}^{⊤},$$

where *X*, *E*, *B*, *C*, and *P* denote biomass concentration, enzyme activity, insoluble substrate, soluble sugar, and ethanol concentration, respectively. The manipulated input vector is

(30) $$u\left(t\right)={\left[\begin{array}{cc} T(t) & \mathrm{pH}(t) \end{array}\right]}^{⊤}.$$

The final batch time $t_{f}$ is also optimized because the best batch duration depends on the product and process characteristics [20,21]. The general multi-objective dynamic optimization problem takes the form

(31) $$\min_{u\left(t\right),t_{f}}J\left(u,t_{f}\right)=\left[J_{\mathrm{EtOH}},J_{\mathrm{prod}},J_{\mathrm{conv}},J_{\mathrm{sugar}},J_{\mathrm{sev}},J_{\Delta u},J_{\mathrm{time}}\right],$$

subject to the dynamic CBP model

(32) $$\begin{array}{cc} \dot{x}\left(t\right) & =f\left(x\left(t\right),u\left(t\right),\theta \right),\;0\leq t\leq t_{f}, \end{array}$$

(33) $$\begin{array}{cc} x(0) & =x_{0}, \end{array}$$

and operating constraints

(34) $$\begin{array}{cc} T_{\min} & \leq T\left(t\right)\leq T_{\max}, \end{array}$$

(35) $$\begin{array}{cc} {\mathrm{pH}}_{\min} & \leq \mathrm{pH}\left(t\right)\leq {\mathrm{pH}}_{\max}, \end{array}$$

(36) $$\begin{array}{cc} t_{f,\min} & \leq t_{f}\leq t_{f,\max}. \end{array}$$

In the main simulation, temperature, pH, and final batch time are confined to the ranges

(37) $$30\leq T\left(t\right)\leq 55,\;5.0\leq \mathrm{pH}\left(t\right)\leq 8.0,\;36\leq t_{f}\leq 144\;h.$$

Feasibility conditions are enforced through dynamic simulation of the system. A policy is deemed feasible if it satisfies both the substrate conversion and sugar accumulation requirements, as follows:

(38) $$\begin{array}{cc} X_{\mathrm{conv}} & \geq X_{\mathrm{conv},\min}, \end{array}$$

(39) $$\begin{array}{cc} C(t) & \leq C_{\max}. \end{array}$$

The feasibility criterion in the main experiments is $X_{\mathrm{conv},\min}=0.42$, while the soft constraint for sugar concentration is $C_{\max}=6\;g\;L^{-1}$. The substrate conversion threshold is further analyzed through sensitivity analysis because feasible conversion is model-specific and depends on kinetic parameters and operating bounds.

### 3.2. Policy Parameterization

The temperature and pH profiles are parameterized using piecewise-constant dynamic policies over $N_{u}$ control intervals. In the main experiment, $N_{u}=16$. Given the final batch time $t_{f}$, the policy grid is

(40) $$0=\tau_{0}<\tau_{1}<\cdots <\tau_{N_{u}}=t_{f},$$

and the control strategy is defined as

(41) $$u\left(t\right)={\left[\begin{array}{cc} T_{j} & {\mathrm{pH}}_{j} \end{array}\right]}^{⊤},\;\tau_{j-1}\leq t<\tau_{j},\;j=1,\ldots ,N_{u}.$$

In this way, each policy candidate is described by

(42) $$\pi =\left[T_{1},\ldots ,T_{N_{u}},{\mathrm{pH}}_{1},\ldots ,{\mathrm{pH}}_{N_{u}},t_{f}\right].$$

Through direct parameterization, the dynamic optimization problem is transformed into a policy-search problem in a finite-dimensional space. This kind of parameterization is common in dynamic optimization because it allows the use of nonlinear operating strategies in an interpretable and computationally tractable form while still preserving the time dependence of the control variables [10]. In this study, the same formulation also supports high-throughput evaluation of feasible and infeasible CBP policies, which is useful for constructing a clear Pareto archive.

### 3.3. Performance Objectives

The performance objectives are computed based on the results of the dynamic simulation from t=0 to $t=t_{f}$. As the problem formulation in Equation (31) implies a minimization problem setup, naturally maximized objectives are scaled by the factor of −1.

#### 3.3.1. Final Ethanol Titer

The ethanol titer objective maximizes the terminal concentration of the ethanol product as follows:

(43) $$J_{\mathrm{EtOH}}=-P\left(t_{f}\right).$$

The ethanol titer represents the traditional batch termination criterion and is included as a benchmark measure to evaluate the performance of alternative high-titer policies.

#### 3.3.2. Volumetric Productivity

The volumetric productivity objective maximizes the ethanol production rate per unit of batch duration as follows:

(44) $$J_{\mathrm{prod}}=-\frac{P\left(t_{f}\right)-P\left(0\right)}{t_{f}}.$$

Because P(0)=0, the function simplifies to $-P\left(t_{f}\right)/t_{f}$. The productivity objective is included to prevent the selection of a high final ethanol titer at the cost of an excessively long batch duration.

#### 3.3.3. Substrate Conversion

The conversion objective maximizes the proportion of the consumed insoluble substrate as follows:

(45) $$J_{\mathrm{conv}}=-X_{\mathrm{conv}}=-\frac{B\left(0\right)-B\left(t_{f}\right)}{B(0)}.$$

The inclusion of the substrate conversion objective prevents the optimization from favoring inefficient policies in terms of the utilization of the available substrates.

#### 3.3.4. Sugar Accumulation

Soluble sugar accumulation is minimized by maximizing the time average of its concentration as follows:

(46) $$J_{\mathrm{sugar}}=\frac{1}{t_{f}}\int_{0}^{t_{f}}C\left(t\right)\;dt.$$

In this objective, a policy generating faster release of soluble sugars compared to the speed of consumption during the fermentation stage is penalized. Sugar accumulation serves as a convenient metric of the imbalance in CBP between the hydrolysis and fermentation stages.

#### 3.3.5. Operating Severity

The thermal and pH severity are minimized compared to mild conditions, as follows:

(47) $$J_{\mathrm{sev}}=\frac{1}{t_{f}}\int_{0}^{t_{f}}\left[{\left(\frac{T\left(t\right)-T_{\mathrm{ref}}}{\Delta T}\right)}^{2}+{\left(\frac{\mathrm{pH}\left(t\right)-{\mathrm{pH}}_{\mathrm{ref}}}{\Delta \mathrm{pH}}\right)}^{2}\right]dt.$$

$T_{\mathrm{ref}}$ and ${\mathrm{pH}}_{\mathrm{ref}}$ are defined as the values representing mild conditions near the preferred region of the nominal CBP process dynamics. Operating severity is included as an objective in order to distinguish between high-performance operating policies requiring aggressive conditions and those providing acceptable results under milder settings.

#### 3.3.6. Control Movement

Aggressive temperature and pH adjustments are discouraged by minimizing the control movement objective, as follows:

(48) $$J_{\Delta u}=\sum_{j=1}^{N_{u}-1}\left[{\left(T_{j+1}-T_{j}\right)}^{2}+{\left({\mathrm{pH}}_{j+1}-{\mathrm{pH}}_{j}\right)}^{2}\right].$$

This penalty favors smoother operating policies for CBP by discouraging large step-to-step changes in the manipulated variables. In practical terms, it helps the optimizer avoid unnecessarily abrupt temperature and pH transitions when a similar ethanol titer, productivity, or conversion can be achieved with gentler profiles.

It should be noted that Equation (48) is a movement penalty rather than a hard actuator constraint. Thus, it does not directly prescribe the maximum heating rate, cooling rate, or pH adjustment rate that can be achieved by a specific reactor system. Those limits are equipment- and scale-dependent and may vary with reactor volume, heat transfer capacity, agitation, buffering behavior, and the acid/base dosing system. Therefore, in the present study, the control-movement objective is used to encourage operational smoothness, while explicit ramp rate constraints are identified as a practical extension for pilot- or plant-scale implementation.

If equipment-specific limits are known, the formulation can be extended by combining Equation (48) with hard rate-of-change constraints, such as those given in Equation (4). This would ensure that the optimized temperature and pH policies are not only smooth in a numerical sense but also compatible with the physical capabilities of the available control hardware.

#### 3.3.7. Batch Duration

Finally, the batch time objective is included to minimize the batch duration, as follows:

(49) $$J_{\mathrm{time}}=t_{f}.$$

The inclusion of $t_{f}$ as an objective variable allows the identification of both shorter and longer optimal batches depending on whether productivity or substrate conversion dominates the optimization problem setup, respectively. Residence time is known as a major source of the conflict between the productivity and conversion objectives.

The seven objective functions used for the Pareto optimality analysis together with the direction of their optimization and physical interpretation are listed in Table 7.

### 3.4. Pareto Optimality and Domination

A dynamic policy $\pi_{a}$ is considered to dominate another dynamic policy $\pi_{b}$ when it is no worse in any objective and strictly better in at least one objective. Using the minimization vector J, the dominance relation is expressed as

(50) $$\pi_{a}≺\pi_{b}\;⟺\;J_{i}\left(\pi_{a}\right)\leq J_{i}\left(\pi_{b}\right)\;\forall i\;\mathrm{and}\;J_{k}\left(\pi_{a}\right)<J_{k}\left(\pi_{b}\right)\;\mathrm{for}\;\mathrm{at}\;\mathrm{least}\;\mathrm{one}\;k.$$

If there is no other feasible policy that dominates a given policy, then that policy is considered Pareto-optimal. The collection of non-dominated feasible policies forms the Pareto set, while their corresponding objective values form the Pareto front. This concept is useful for analyzing CBP operation because a single scalar criterion can obscure important process trade-offs. For example, maximizing the ethanol titer may require a longer batch time, whereas maximizing productivity may favor earlier termination with a lower final ethanol concentration. Similar trade-offs have motivated Pareto-based formulations in fed-batch bioreactor optimization and dynamic bioprocess optimal control [10,22].

### 3.5. Feasibility-Aware Pareto Front Generation

The Pareto front was generated using a feasibility-aware archive-based direct dynamic policy-search algorithm. This method is not an off-the-shelf implementation of NSGA-II, SPEA2, or another standard evolutionary multi-objective optimizer. Instead, it uses the central idea of Pareto dominance and NSGA-II-style non-dominated sorting [12], but applies these concepts within a custom archive-based policy-search framework designed for the CBP dynamic optimization problem.

Each candidate solution represents a complete CBP operating policy. A policy contains the piecewise-constant temperature profile, the piecewise-constant pH profile, and the final batch time. Thus, each policy can be written as

(51) $$\pi =\left[T_{1},\ldots ,T_{N_{u}},{\mathrm{pH}}_{1},\ldots ,{\mathrm{pH}}_{N_{u}},t_{f}\right].$$

For every candidate policy, the CBP dynamic model is simulated from t=0 to $t=t_{f}$. The ethanol titer, productivity, substrate conversion, soluble sugar accumulation, operating severity, control movement, and batch time are then computed from the simulated trajectories.

The procedure is feasibility-aware because feasibility screening is performed before the final Pareto set is selected. A policy is classified as feasible if it satisfies the minimum conversion requirement and the sugar accumulation constraint. When feasible policies are available, non-dominated sorting is applied only to the feasible archive. This ensures that the reported Pareto set represents operating strategies that satisfy the prescribed process constraints, rather than policies that perform well on one objective but violate the feasibility requirements.

Feasibility awareness is implemented as a separate post-simulation screening and archive-filtering step, rather than as a penalty term added to the objective vector. Each candidate policy is first simulated, and the objective values are computed from the resulting trajectories. The feasibility conditions are then evaluated separately using a binary feasibility flag as follows:

(52) $$F\left(\pi \right)=\left\{\begin{array}{cc} 1, & X_{\mathrm{conv}}\left(\pi \right)\geq X_{\mathrm{conv},\min}\;\mathrm{and}\;C\left(t;\pi \right)\leq C_{\max},\\ 0, & \mathrm{otherwise}. \end{array}\right.$$

Here, F(π)=1 denotes a feasible policy, and F(π)=0 denotes an infeasible policy. The feasibility flag is not included as a weighted loss term, and infeasible policies are not converted into feasible policies through soft penalties. Instead, when feasible policies are available, the main non-dominated sorting step is applied only to the feasible subset of the archive. Infeasible policies are still retained in the full archive for diagnostic purposes, including feasibility threshold sensitivity analysis, but they are not included in the reported feasible Pareto set. Thus, the term “feasibility-aware” refers to explicit feasibility screening before Pareto front extraction rather than to a scalar penalty function embedded in the multi-objective loss.

The algorithm starts by generating an initial archive of random dynamic policies within the admissible temperature, pH, and batch-time bounds. Each policy is simulated and evaluated. Feasible non-dominated policies are then identified and used to guide the generation of new candidate policies. New policies are produced by perturbing and recombining high-performing archive members. The simulation, feasibility screening, archive update, and non-dominated sorting steps are repeated for a prescribed number of generations. The final archive contains all evaluated policies, while the reported Pareto front is extracted from the feasible non-dominated subset.

The feasibility-aware policy-search procedure is summarized in Table 8.

This algorithm is conceptually related to NSGA-II because it uses Pareto dominance and non-dominated sorting [12]. However, it does not implement the full standard NSGA-II workflow, such as tournament selection, crowding-distance-based survival, and standard crossover and mutation operators, exactly as defined in the original algorithm. It is also different from SPEA2 because it does not use the SPEA2 strength–fitness assignment and external archive update mechanism. The present implementation is better described as a custom feasibility-aware archive-based direct policy search.

This choice was made because the CBP optimization problem has several features that make a direct policy-search archive convenient: nonlinear dynamic state equations, free terminal batch time, explicit feasibility thresholds, and multiple objectives with different physical units. Scalarization methods, such as weighted sums, are simple and widely used, but they can miss nonconvex regions of the Pareto front. Normal boundary intersection provides a structured way to sample trade-off surfaces [13], while the normalized normal constraint method improves Pareto front representation in multi-objective design problems [14]. In dynamic bioprocess optimization, direct optimal control and Pareto generation approaches have also been used to construct trade-off sets for nonlinear systems [10]. The archive-based method used here is therefore appropriate for screening a large number of nonlinear CBP operating policies while retaining both feasible and infeasible policy information for later feasibility and sensitivity analysis.

### 3.6. Representative Policy Selection

Once the feasible Pareto set is obtained, representative policies are selected to make the trade-offs easier to interpret. The following six extreme Pareto policies are chosen by optimizing one objective at a time within the feasible Pareto set:

(53) $$\begin{array}{cc} \pi_{\mathrm{EtOH}} & =\arg\max_{\pi \in P}P\left(t_{f}\right), \end{array}$$

(54) $$\begin{array}{cc} \pi_{\mathrm{prod}} & =\arg\max_{\pi \in P}\frac{P\left(t_{f}\right)-P\left(0\right)}{t_{f}}, \end{array}$$

(55) $$\begin{array}{cc} \pi_{\mathrm{conv}} & =\arg\max_{\pi \in P}X_{\mathrm{conv}}, \end{array}$$

(56) $$\begin{array}{cc} \pi_{\mathrm{sugar}} & =\arg\min_{\pi \in P}J_{\mathrm{sugar}}, \end{array}$$

(57) $$\begin{array}{cc} \pi_{\mathrm{sev}} & =\arg\min_{\pi \in P}J_{\mathrm{sev}}, \end{array}$$

(58) $$\begin{array}{cc} \pi_{\mathrm{time}} & =\arg\min_{\pi \in P}t_{f}, \end{array}$$

where P is the feasible Pareto set. These extreme policies are not necessarily recommended operating points. Rather, they are used to reveal the performance limits associated with each individual process priority.

A balanced-knee Pareto policy is also selected as a practical compromise when no single objective is intended to dominate the decision. Before selecting this policy, the objectives are normalized so that larger values indicate better performance. The policy whose normalized objective vector is closest to the ideal normalized point is then chosen as follows:

(59) $$\pi_{\mathrm{knee}}=\arg\min_{\pi \in P}{∥z^{*}-z\left(\pi \right)∥}_{2},$$

where $z^{*}$ is the ideal normalized objective vector and z(π) is the normalized performance vector of policy π. This balanced-knee selection provides a useful compromise solution when the decision requires simultaneous consideration of titer, productivity, conversion, severity, smoothness, sugar accumulation, and batch duration.

### 3.7. Baseline, Sensitivity, and Repeatability Analyses

Three additional analyses are included to support interpretation of the Pareto results. First, static baseline policies are evaluated using constant temperature, constant pH, and fixed batch times. This baseline is used to determine whether dynamic operation provides a meaningful advantage over constant-operation schedules. Such comparisons are important because dynamic control policies should be evaluated against simpler alternatives before being proposed as process-design tools.

Second, sensitivity to the minimum acceptable conversion threshold is examined because this threshold directly determines whether a simulated policy is classified as feasible. This analysis is important because feasibility thresholds in model-based bioprocess optimization depend partly on the assumed kinetic parameters, operating bounds, and substrate representation. The thresholds considered were

(60) $$X_{\mathrm{conv},\min}\in \left\{0.35,\;0.38,\;0.40,\;0.42,\;0.44,\;0.55\right\}.$$

For each threshold, the evaluated policy archive was re-screened using the same ethanol and sugar accumulation requirements. The number of feasible policies, the number of feasible Pareto policies, and the best attainable ethanol titer, productivity, conversion, severity, and batch time were then recalculated. This analysis was used to determine whether the main feasibility threshold was overly permissive, scientifically restrictive but attainable, or too strict to be reached under the present model and operating bounds.

The present sensitivity analysis focuses on the feasibility definition rather than on full kinetic-parameter uncertainty propagation. This choice was made because the kinetic constants used in the reduced-order CBP model are nominal literature-informed values and were not estimated from a dedicated experimental dataset in the present study. Therefore, assigning probability distributions to the kinetic parameters would introduce additional assumptions that are not directly supported by the available data. Nevertheless, the parameters most likely to influence the predicted Pareto policies can be identified from the model structure, including the maximum hydrolysis coefficient $V_{h,\max}$, the maximum ethanol production coefficient $Y_{P,\max}$, the ethanol inhibition coefficient $k_{I}$, the sugar consumption coefficient $k_{C}$, the biomass carrying capacity $K_{X}$, and the temperature–pH activity centers. These parameters should be prioritized in future local sensitivity, uncertainty-propagation, and robust Pareto optimization studies.

Third, independent-seed repeatability runs are performed to check whether the main performance envelope is an artifact of a single random initialization. Stochastic multi-objective searches can produce different numbers of feasible and non-dominated policies across seeds, even when the best attainable performance region remains similar. The repeatability analysis therefore evaluates whether the main conclusions are robust to stochastic search variation.

### 3.8. Positioning Relative to Alternative Optimization and Control Frameworks

The present method is best interpreted as an offline feasibility-aware direct policy-search framework rather than as a standard evolutionary optimizer, online controller, or learning-based control algorithm. This distinction is important when comparing the proposed approach with advanced alternatives such as NSGA-II, model predictive control (MPC), and reinforcement learning (RL).

NSGA-II and related evolutionary multi-objective algorithms provide well-established procedures for non-dominated sorting, population evolution, and diversity preservation through crowding distance [12]. The present method uses Pareto dominance and non-dominated sorting, but it does not implement the full NSGA-II workflow. Instead, it uses a custom archive-based direct search over complete temperature–pH–time policies and applies feasibility screening before extracting the reported Pareto front. This makes the method simpler to connect with the reduced-order CBP model and allows infeasible policies to be retained for diagnostic analysis. However, a formal head-to-head comparison with standard NSGA-II or SPEA2 would require implementing the same policy encoding, feasibility rules, search budget, and objective definitions for each algorithm.

MPC provides a different type of framework. Rather than generating an offline Pareto set, MPC repeatedly solves an optimization problem online as new process measurements become available. Such an approach would be attractive for CBP because temperature and pH profiles could be updated during the batch using measured sugar, ethanol, biomass, or substrate indicators. However, MPC requires a sufficiently calibrated dynamic model, online state estimation, and reliable measurements during operation [5,10]. These requirements are beyond the scope of the present in silico policy-screening study, but the representative Pareto policies obtained here could provide initial trajectories, reference schedules, or constraint-informed warm starts for future MPC implementation.

RL-based methods also offer a possible route for adaptive CBP control because they can learn operating policies through repeated interaction with a simulated or experimental environment. However, RL generally requires a large number of training episodes, careful reward design, and validation to ensure physically realistic and safe policies. For CBP, this would require a reliable simulation environment or extensive experimental data, both of which are not yet available for the present reduced-order model. Therefore, RL is better viewed as a future extension once experimentally calibrated dynamics and online measurements become available.

The comparison in Table 9 summarizes the role of these alternative approaches relative to the present framework. The main benchmarking in this study is therefore performed against static constant-operation policies, conversion threshold sensitivity, and independent-seed repeatability. Advanced algorithmic benchmarking against NSGA-II, MPC, and RL is an important future direction, but it should be performed only after harmonizing model assumptions, objective definitions, feasibility constraints, and computational budgets.

### 3.9. Numerical Implementation and Computational Reproducibility

For high-throughput evaluation, the dynamic model was integrated using fixed-step numerical simulation. In the main run, 16,000 initial policies were generated, followed by 14 generations with 10,000 offspring policies per generation. This produced 156,000 candidate policy evaluations, from which a maximum archive of 120,000 policies was retained for post-processing and Pareto analysis. Each candidate policy was described by 16 temperature segments, 16 pH segments, and one terminal-time decision.

The complete computational workflow required approximately 1.6min (1min37s) on the workstation used for this study. Candidate policy scoring was GPU-accelerated when available, while data handling, Pareto sorting, static baseline analysis, conversion threshold sensitivity analysis, independent-seed repeatability summaries, table export, and figure generation were performed on the CPU. This runtime is hardware- and implementation-dependent, but it provides an approximate indication of the computational cost of evaluating a large dynamic policy archive.

Search progress was monitored at the generation level using the number of evaluated policies, the number of feasible policies, the best attainable ethanol titer, the best substrate conversion, and the size of the feasible non-dominated archive. These quantities were used as practical convergence indicators for the stochastic direct policy search. Because the method is archive-based and simulation-driven, convergence was assessed by stabilization of the high-performing region of the feasible Pareto archive rather than by a gradient-based optimality tolerance. The independent-seed repeatability analysis was also used as an additional check that the main high-performing ethanol titer and conversion regions were not artifacts of a single random initialization.

To support computational reproducibility, the workflow was implemented in Python 3.13.5 and developed in Visual Studio Code 1.120.0. The main stochastic Pareto search used a fixed random seed of 42, while the repeatability analysis used independent additional seeds. The computational workflow used NumPy 2.3.2 for vectorized numerical operations, pandas 2.3.1 for tabular data handling, Matplotlib 3.10.3 for figure generation, and OpenPyXL-supported Excel export. Optional GPU acceleration was provided through CuPy, with Torch used only to expose CUDA library paths on compatible Windows installations.

**Figure 1:**
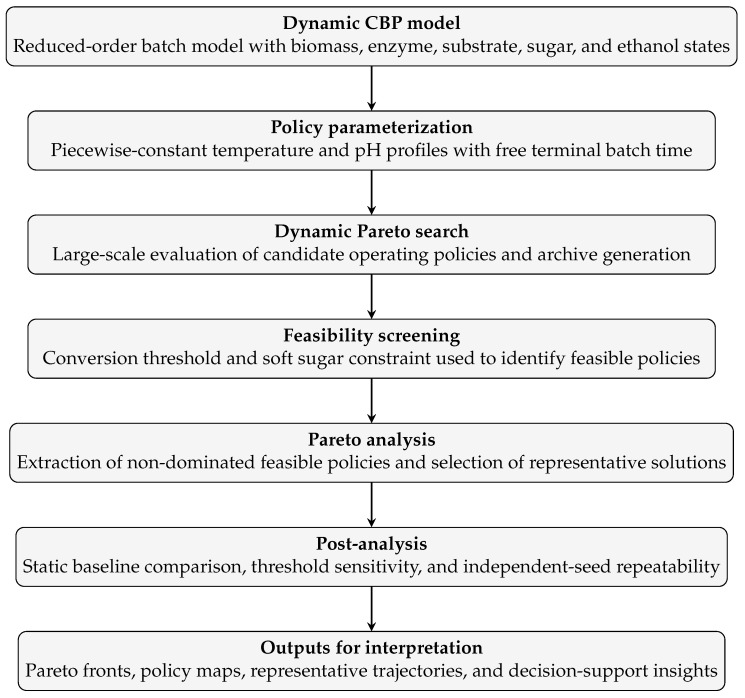
Workflow of the feasibility-aware multi-objective dynamic optimization framework for consolidated bioprocessing.

The full computational workflow, from dynamic CBP modeling and policy generation to feasibility-aware Pareto analysis and post-optimization interpretation, is summarized in Figure 1.

## 4. Results and Discussion

### 4.1. Optimization Scale, Feasibility, and Static Baseline Comparison

The dynamic Pareto optimization retained 120,000 candidate operating policies for post-processing across the batch horizon. Under the main feasibility definition, which required a minimum substrate conversion of $X_{\mathrm{conv},\min}=0.42$, 5017 policies were classified as feasible. This represents 4.18% of the retained archive. From this feasible subset, 328 policies were retained in the main non-dominated Pareto set, corresponding to 6.54% of the feasible policies and 0.27% of the full retained archive. These values show that the feasibility and Pareto filters were selective while still preserving a sufficiently large set of trade-off solutions for interpretation.

The feasible Pareto set covered a broad range of operating priorities, including high ethanol titer, high productivity, high substrate conversion, low sugar accumulation, low operating severity, low control movement, and a short batch duration. Within the feasible Pareto archive, the highest ethanol titer was $1.265\;g\;L^{-1}$, the highest substrate conversion was 0.440, the lowest severity value was 0.027, and the shortest feasible batch time was 51.46h. The coexistence of these different optima indicates that the feasible archive did not collapse into a single operating style. Instead, it retained distinct policy types that reflect different process priorities.

The ethanol titers predicted in this study are low compared to the values generally required for industrially competitive ethanol recovery. Published CBP studies and reviews commonly identify a high ethanol titer and ethanol tolerance as major requirements for commercial viability, with targets often discussed at or above about $40\;g\;L^{-1}$ to reduce downstream separation costs and improve process economics [3,4,23]. The maximum titer obtained here, $1.265\;g\;L^{-1}$, is only about 3.2% of a $40\;g\;L^{-1}$ reference target. Therefore, the absolute ethanol titers should not be interpreted as industrially sufficient process outcomes.

The simulated titer is also lower than the values reported for the higher-performing CBP strain and process development studies. Improved *Clostridium thermocellum* systems have been reported to achieve substantially higher ethanol yields and titers under optimized conditions [24], and engineered or evolved *C. thermocellum* strains have been studied for CBP of lignocellulosic feedstocks such as switchgrass [25]. The present model should therefore be understood as a nominal reduced-order virtual plant for comparing operating policies rather than as a calibrated high-titer industrial CBP configuration. Its practical value lies in showing how dynamic temperature and pH policies alter relative performance, feasibility, and trade-off structure under fixed model assumptions. Translation to industrially relevant CBP would require recalibration at higher substrate loading, improved strain performance, inclusion of feedstock heterogeneity and co-product formation, validation with time-resolved experimental data, and assessment of downstream recovery and scale-up constraints.

The conversion threshold sensitivity analysis showed that the feasible policy archive depends strongly on the selected minimum conversion requirement. Lower thresholds, such as $X_{\mathrm{conv},\min}=0.35$, 0.38, and 0.40, expanded the feasible region and allowed more policies to be retained for Pareto analysis. The main threshold of $X_{\mathrm{conv},\min}=0.42$ was more restrictive, but it remained attainable. When the threshold was increased further, the feasible region narrowed substantially; thresholds of $X_{\mathrm{conv},\min}=0.44$ and 0.55 were not attainable under the current reduced-order model, operating bounds, and evaluated archive. This places the main threshold close to the upper attainable conversion region of the present simulation framework. Thus, $X_{\mathrm{conv},\min}=0.42$ functions as a stringent substrate-utilization filter for this in silico CBP model, not as an experimentally established industrial benchmark for all CBP systems.

To assess the benefit of dynamic operation, a static baseline grid was evaluated using constant temperature, constant pH, and different batch termination times. The term “best static policy” refers to the best-performing constant-operation policy selected independently for each performance criterion from the static grid. Therefore, the best static policy for the ethanol titer is not necessarily the same as the best static policy for productivity or conversion. For ethanol titer, productivity, and conversion, the best static baselines were $1.144\;g\;L^{-1}$, $0.0157\;g\;L^{-1}\;h^{-1}$, and 0.384, respectively. The corresponding dynamic Pareto values were $1.265\;g\;L^{-1}$, $0.0170\;g\;L^{-1}\;h^{-1}$, and 0.440. These differences correspond to improvements of 10.6%, 8.3%, and 14.3%, respectively, as shown in Table 10.

**Figure 2:**
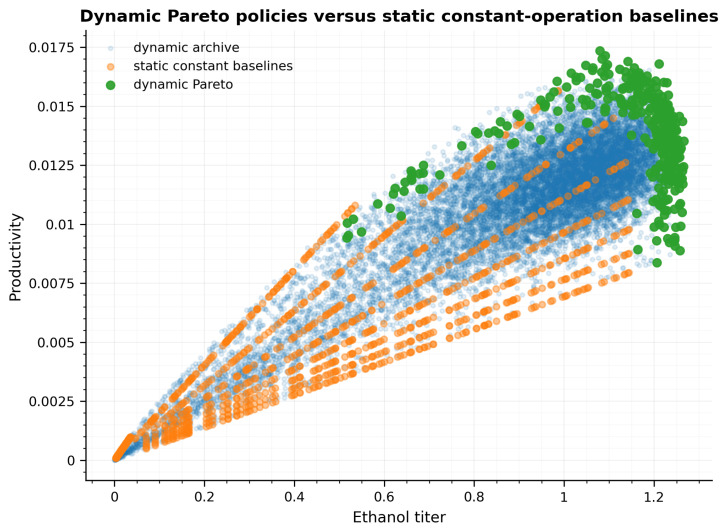
Comparison of dynamic policy candidates, static constant-operation baselines, and feasible dynamic Pareto policies in ethanol–productivity objective space. The quantitative comparison in Table 10 shows that the dynamic Pareto policies improve ethanol titer, productivity, and substrate conversion by 10.6%, 8.3%, and 14.3%, respectively, relative to criterion-specific best static baselines.

None of the criterion-specific static baseline policies satisfied the main conversion feasibility threshold. The static baselines are therefore interpreted as unconstrained constant-operation benchmarks rather than feasible operating strategies under the main constraint definition. Even with this conservative interpretation, the quantitative comparison shows that dynamic temperature and pH scheduling expands the upper performance envelope relative to constant-operation policies. The ethanol–productivity plot supports this conclusion visually: static baselines occupy a lower productivity–titer region, whereas the feasible dynamic Pareto policies extend toward higher ethanol and productivity values, as shown in Figure 2.

### 4.2. Biochemical Interpretation of Dynamic Temperature and pH Policies

The optimized temperature and pH trajectories can be interpreted as staged operating hypotheses for CBP rather than simply as numerical optimization outputs. In the early part of the batch, policies that remain close to the nominal growth and enzyme formation region support biomass development and cellulase activity. This early support is important because cellulolytic organisms such as *Clostridium thermocellum* must establish sufficient biological and enzymatic capacity before substantial substrate solubilization can occur [3,8]. Thus, early operation that favors growth and enzyme formation increases the potential for later hydrolysis.

During the intermediate part of the batch, several high-performing policies shift toward hydrolysis-favorable conditions. This behavior reflects the need to convert an insoluble cellulose-rich substrate into soluble sugars before those sugars can be fermented to ethanol. In CBP, hydrolysis and fermentation occur in the same reactor, but they may not proceed at the same rate. If hydrolysis temporarily exceeds sugar consumption, soluble sugars can accumulate; if fermentation dominates too early, ethanol production may become sugar-limited. The dynamic policies therefore represent a balance between releasing soluble sugars and avoiding excessive sugar buildup. This interpretation is consistent with CBP studies showing that cellulose solubilization, cellulase activity, and fermentation are tightly coupled [7,26].

In the later part of the batch, policies that favor ethanol formation generally move toward conditions associated with fermentation performance and reduced hydrolysis emphasis. Biochemically, this corresponds to a shift from building hydrolytic capacity and releasing soluble sugars to consuming available sugars and accumulating ethanol. At this stage, ethanol inhibition becomes more relevant because increasing product concentration can reduce effective microbial activity and fermentation performance [8,16].

The temperature and pH trajectories should not be interpreted as evidence that the microorganism experiences completely separate biological phases. Growth, enzyme formation, hydrolysis, fermentation, and inhibition overlap throughout CBP operation. Instead, the trajectories indicate which subprocess is emphasized by the operating policy at different parts of the batch. This distinction is important because a single constant temperature and pH condition is unlikely to be ideal for cellulase activity, microbial growth, sugar release, ethanol production, and inhibition management at the same time. The biochemical significance of the Pareto policies is therefore that they provide interpretable strategies for managing these competing requirements within a single-reactor CBP configuration.

### 4.3. Many-Objective Pareto Structure

The many-objective structure of the feasible Pareto set is summarized in Table 11. The final feasible Pareto set contained 328 non-dominated policies, satisfying the primary feasibility criterion $X_{\mathrm{conv}}\geq 0.42$. Across this set, ethanol titer ranged from 0.517 to $1.265\;g\;L^{-1}$, productivity ranged from 0.00838 to $0.01696\;g\;L^{-1}\;h^{-1}$, and substrate conversion ranged from 0.420 to 0.4395. These ranges show that the feasible Pareto set retained meaningful variation in process performance even after applying the conversion constraint.

The dispersion in the minimization objectives further illustrates the trade-off structure. Average sugar accumulation varied from 0.236 to $0.578\;g\;L^{-1}$, operating severity from 0.027 to 0.140, control movement from 0.0038 to 0.1436, and batch time from 51.5 to 143.9h. The batch-time range was especially important because the longest Pareto policies were approximately 2.8 times longer than the shortest feasible Pareto policy. This indicates that some policies achieved a higher ethanol titer or conversion by extending the residence time, whereas shorter policies improved cycle-time performance but sacrificed some final conversion or titer.

The median values also help interpret the central tendency of the Pareto set. The median ethanol titer was $1.203\;g\;L^{-1}$, the median productivity was $0.01418\;g\;L^{-1}\;h^{-1}$, and the median conversion was 0.428. These values are close to the upper end of the feasible conversion range, showing that most retained Pareto policies satisfied the conversion threshold only within a narrow margin. In contrast, severity, movement, and batch time spanned wider relative ranges, indicating that operating smoothness and mildness are major sources of policy differentiation.

Overall, the quantitative spread in Table 11 confirms that no single operating policy simultaneously optimizes all objectives. Policies that favor a high ethanol titer and substrate conversion generally require longer batch durations or accept higher sugar accumulation, whereas policies that prioritize short batch time, low severity, or low control movement sacrifice part of the titer or conversion performance. This reinforces the central conclusion that CBP operation is characterized by structured trade-offs rather than by a single universally optimal operating point. Such an interpretation is consistent with the Pareto analysis in the multi-objective process optimization, where the goal is to reveal decision-relevant compromises rather than collapse them into one scalar optimum [10,12].

### 4.4. Distribution of the Evaluated Policy Archive

Before extracting the feasible Pareto set, the full evaluated archive was examined to assess the breadth of the dynamic policy search. The retained archive contained 120,000 candidate policies, from which 5017 feasible policies and 328 feasible Pareto policies were obtained under the main feasibility definition. The archive-level distributions therefore provide context for understanding how selective the feasibility and Pareto filters were.

**Figure 3:**
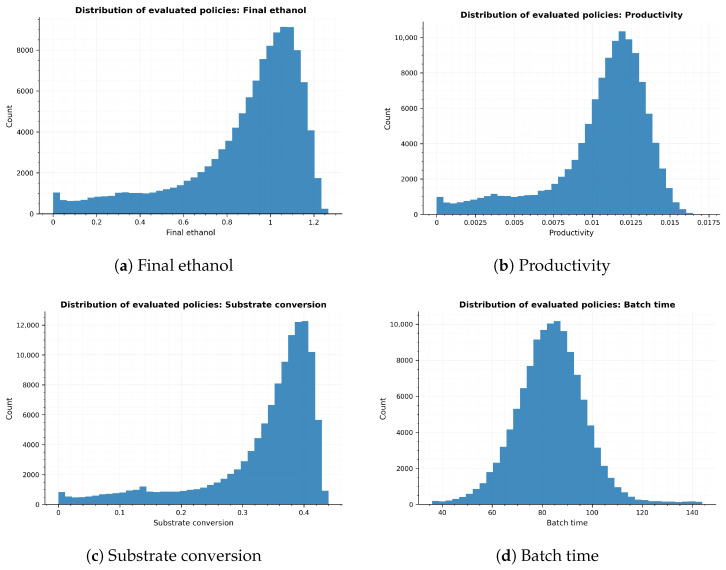
Distributions of key performance metrics across the evaluated dynamic-policy archive before feasible Pareto filtering.

The distributions in Figure 3 show that the search covered a broad operating region rather than a narrow local neighborhood. Final ethanol and productivity were concentrated toward the upper, higher-performing part of the simulated range, while conversion values approached the imposed feasibility boundary of $X_{\mathrm{conv},\min}=0.42$. Batch times covered both shorter high-productivity policies and longer high-conversion policies, indicating that the search explored different residence-time strategies. This archive-level spread supports the subsequent Pareto analysis because the non-dominated set was extracted from a diverse candidate population rather than from a narrowly constrained search space [10,12].

### 4.5. Representative Pareto-Optimal Policies

To improve the interpretability of the feasible Pareto set, representative policies were extracted to reflect different process priorities. The maximum-ethanol policy achieved $1.265\;g\;L^{-1}$ ethanol at a batch time of 101.7h, whereas the maximum-productivity policy shortened the batch to 68.3h and achieved $0.0170\;g\;L^{-1}\;h^{-1}$. This represents a 32.8% reduction in batch time relative to the maximum-ethanol policy, but with a lower final ethanol titer of $1.158\;g\;L^{-1}$. Thus, the productivity-optimal policy gains time efficiency by accepting an approximately 8.5% reduction in final ethanol titer.

The maximum-conversion policy reached the highest substrate conversion, 0.440, while producing $1.214\;g\;L^{-1}$ ethanol at 91.8h. This indicates that maximizing conversion does not necessarily maximize ethanol titer because additional substrate utilization may also be associated with different sugar accumulation, inhibition, and time effects. In contrast, the minimum-sugar policy achieved the lowest average sugar accumulation, $0.236\;g\;L^{-1}$, but required the longest batch duration, 143.9h. This suggests that minimizing soluble sugar buildup can require longer operation so that fermentation can consume the released sugar more completely.

The balanced-knee policy provides a useful compromise among the competing objectives. It achieved $1.216\;g\;L^{-1}$ ethanol, $0.0145\;g\;L^{-1}\;h^{-1}$ productivity, and 0.434 conversion at 83.8h. Relative to the maximum-ethanol policy, the balanced-knee policy reduced batch time by 17.6h while maintaining 96.1% of the maximum ethanol titer. Relative to the maximum-conversion policy, it retained 98.6% of the maximum conversion. These quantitative comparisons show why the balanced-knee policy is more attractive for decision support than any single extreme policy. The representative policy summary is provided in Table 12.

### 4.6. Pairwise Pareto Trade-Offs

**Figure 4:**
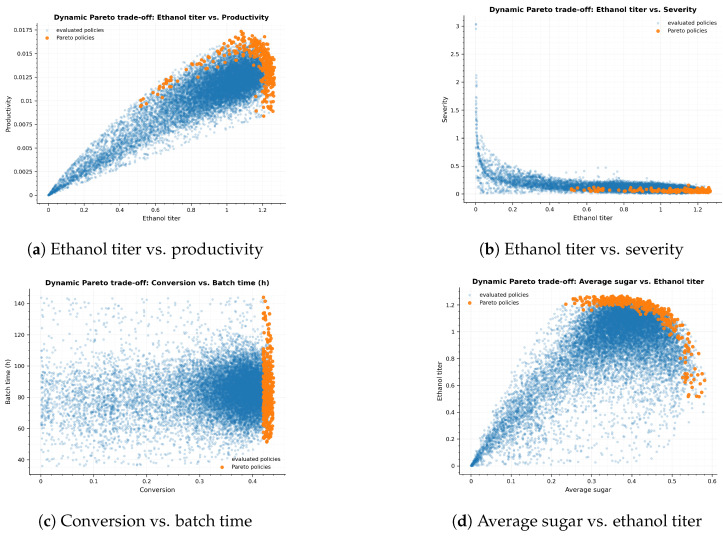
Pairwise Pareto trade-offs for feasible dynamic CBP operating policies.

The pairwise Pareto plots show how the objectives interact within the feasible operating region and summarize the trade-offs among ethanol titer, productivity, severity, conversion, batch time, and sugar accumulation (Figure 4). In the ethanol–productivity plane, the highest ethanol titer and highest productivity are achieved by different policies (Figure 4a). The maximum-ethanol policy reached $1.265\;g\;L^{-1}$ but only $0.0124\;g\;L^{-1}\;h^{-1}$, whereas the maximum-productivity policy reached $0.0170\;g\;L^{-1}\;h^{-1}$ with a lower ethanol titer of $1.158\;g\;L^{-1}$. This confirms that final-product accumulation and time-normalized productivity are related but not interchangeable objectives. Such tension between terminal performance and productivity is common in dynamic bioprocess optimization [10].

The ethanol–severity relationship indicates that high ethanol production does not always require the most severe operating conditions (Figure 4b). For example, the minimum-severity policy achieved $1.255\;g\;L^{-1}$ ethanol with a severity value of 0.027, which is 99.2% of the maximum ethanol titer, while using the lowest severity among the representative policies. This suggests that mild temperature–pH trajectories can still approach the upper ethanol region in the present model. From a process-design perspective, this is important because lower severity may reduce operational stress, improve implementability, and simplify scale-up.

The conversion–batch time relationship shows a clearer trade-off (Figure 4c). The maximum-conversion policy achieved 0.440 conversion at 91.8h, whereas the minimum-batch-time policy ended at 51.5h and produced a much lower ethanol titer of $0.518\;g\;L^{-1}$. Although the minimum-batch-time policy still satisfied the conversion constraint, its lower ethanol performance shows that shortening the batch too strongly can reduce product accumulation. This supports the interpretation that additional residence time is useful only when it produces meaningful gains in conversion or ethanol formation. Similar trade-offs between process performance and operation time have been reported in Pareto analyses of bioreactor operation [22].

The sugar–ethanol relationship reflects the balance between hydrolysis and fermentation (Figure 4d). Higher ethanol-producing policies generally tolerate moderate soluble sugar accumulation, while the minimum-sugar policy reduces average sugar to $0.236\;g\;L^{-1}$ but requires 143.9h. This indicates that minimizing sugar accumulation is not equivalent to maximizing ethanol or productivity. Instead, some intermediate sugar availability is beneficial because it reflects substrate hydrolysis supplying fermentable sugar for ethanol production. Overall, the pairwise plots confirm that CBP operation requires choosing among competing priorities rather than selecting a single universally best policy [12].

### 4.7. Dynamic Operating Policy Maps

**Figure 5:**
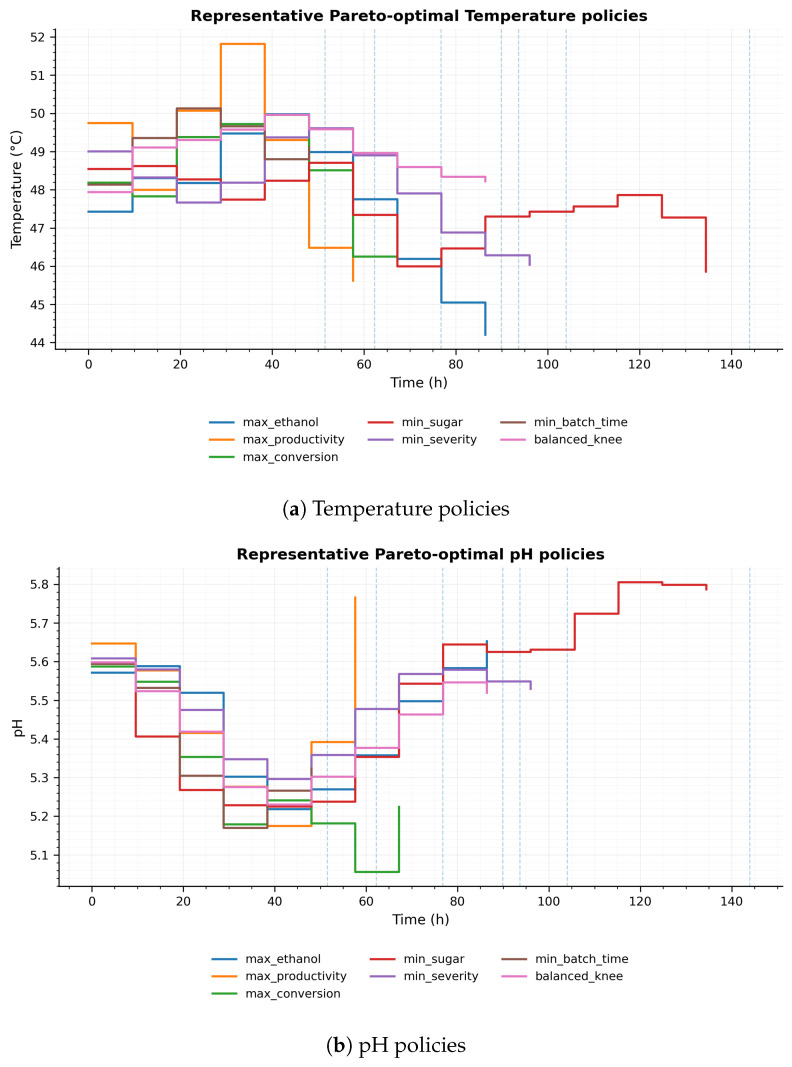
Representative dynamic temperature and pH policies selected from the feasible Pareto set.

Representative temperature and pH profiles show how the selected feasible Pareto policies translate objective-space trade-offs into implementable operating schedules. Most high-performing policies operate within a relatively narrow window of approximately 47–50 °C and pH 5.2–5.9, corresponding to a temperature span of about 3 °C and a pH span of about 0.7 units for the main high-performing operating region. Thus, improved CBP performance in the present model is not associated with erratic or extreme control behavior, but with moderate time-dependent adjustments inside a biologically favorable window, as shown in Figure 5.

Differences among the representative policies are nevertheless clear. The high-ethanol and high-conversion policies maintain moderately elevated temperatures during the hydrolysis-dominant part of the batch before shifting toward milder late-stage conditions. In contrast, the minimum-severity and balanced-knee policies remain closer to the nominal preferred operating region for most of the batch. These smoother policies are important because they suggest that strong performance can be approached without large temperature or pH excursions. For example, the minimum-severity representative policy still achieves $1.255\;g\;L^{-1}$ ethanol, which is 99.2% of the maximum ethanol titer, while giving the lowest severity value among the representative policies. This supports the practical interpretation that mild dynamic operation can remain competitive with more performance-oriented schedules.

**Figure 6:**
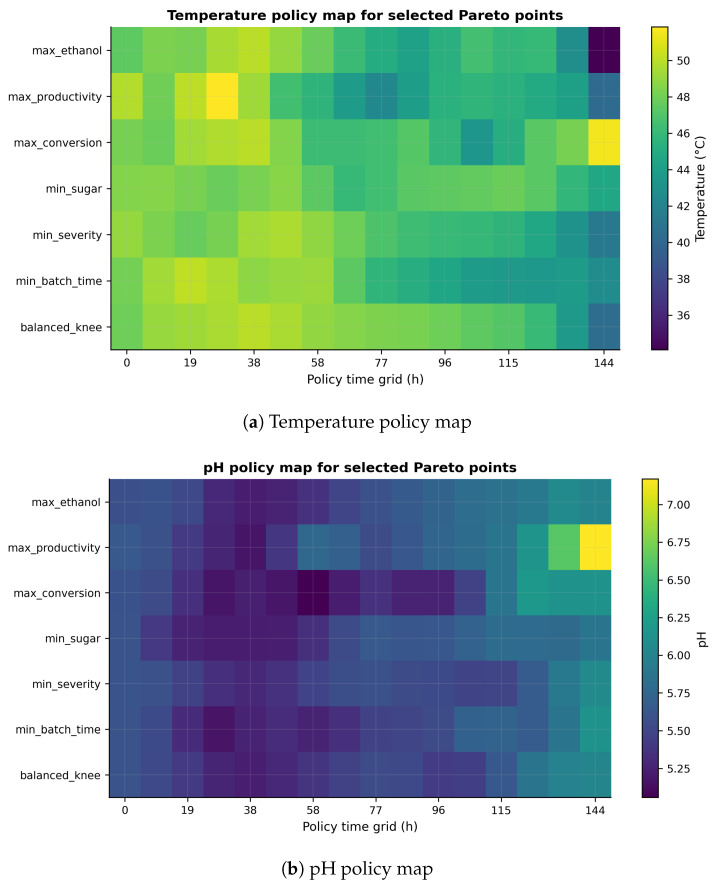
Operating policy maps for representative Pareto-optimal CBP policies.

The policy maps provide a compact view of these operating strategies across the full time grid. They show how different decision priorities are translated into different temporal patterns. Policies favoring ethanol titer or conversion tend to preserve longer hydrolysis- and fermentation-supporting operation, whereas policies favoring low severity or balanced performance avoid strong deviations from the preferred temperature and pH region. These maps therefore complement the objective-space tables by showing not only which policies perform well but also how the reactor would need to be operated to obtain those outcomes, as shown in Figure 6.

### 4.8. State Trajectories Under Representative Pareto Policies

The representative state trajectories clarify how the selected Pareto policies balance substrate hydrolysis, soluble sugar availability, and ethanol formation over time. In CBP, cellulose solubilization, sugar release, microbial growth, fermentation, and product inhibition occur in the same reactor and are strongly coupled [3,7]. For this reason, the timing of ethanol formation, sugar accumulation, and substrate depletion helps explain why different policies favor different objectives.

**Figure 7:**
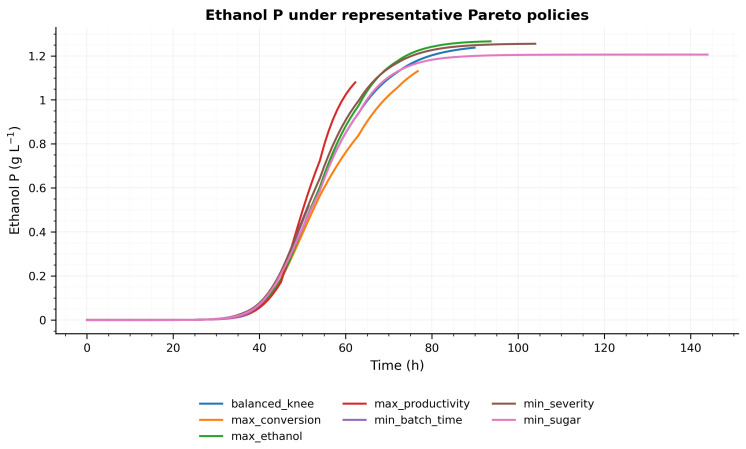
Ethanol trajectories for representative feasible Pareto-optimal policies. The delayed increase in ethanol reflects the model behavior in which fermentable sugar must first become available through substrate hydrolysis before ethanol production becomes dominant.

Ethanol formation remains limited during the early batch period and increases more clearly after sufficient soluble sugar has been generated. The stronger ethanol-producing policies reach approximately 1.2–$1.27\;g\;L^{-1}$ by the end of the batch. This trajectory shape is consistent with batch CBP behavior, where ethanol production depends on the availability of fermentable sugars released from an insoluble cellulose-rich substrate. Kinetic studies of *Clostridium thermocellum* similarly describe ethanol formation as coupled to growth, substrate utilization, and inhibition effects rather than as an isolated endpoint response [8,16]. Although the absolute titers remain below industrially relevant targets, the trajectory comparison is useful for showing how the dynamic policies shift the timing and rate of product formation, as shown in Figure 7.

**Figure 8:**
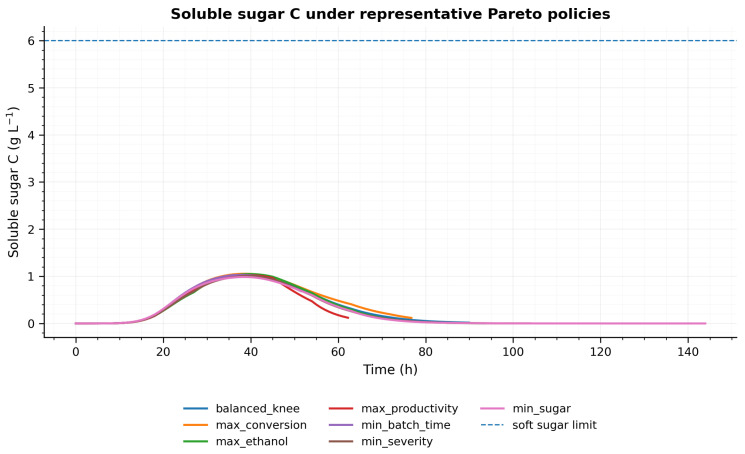
Soluble sugar trajectories for representative feasible Pareto-optimal policies. The mid-batch sugar accumulation followed by a decline indicates a shift from hydrolysis-dominant behavior toward fermentation-dominant behavior.

Soluble sugar increases during the middle part of the batch and then declines as fermentation becomes more dominant. This pattern reflects the hydrolysis–fermentation balance in the reduced-order model. When hydrolysis temporarily exceeds sugar consumption, the soluble sugar pool increases; when fermentation catches up, the pool decreases. All representative policies remain below the imposed sugar threshold, showing that the selected Pareto policies avoid excessive sugar buildup while still supporting ethanol formation. This is consistent with detailed CBP modeling studies showing that saccharification and fermentation are coordinated processes and that mismatch between them can affect product formation and process efficiency [7,26], as shown in Figure 8.

**Figure 9:**
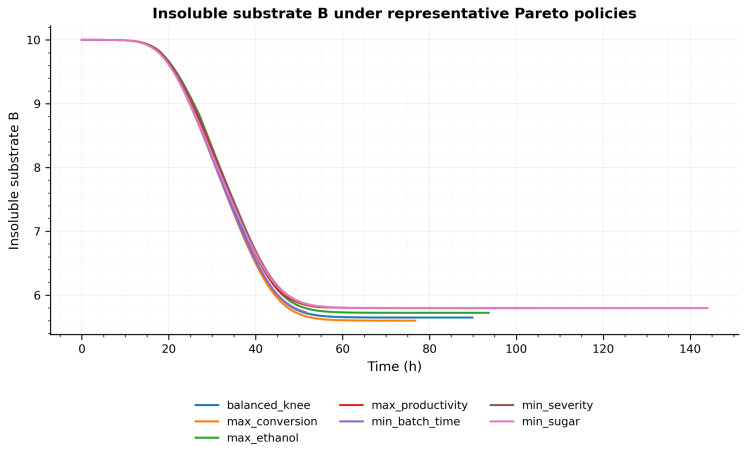
Insoluble substrate trajectories for representative feasible Pareto-optimal policies. Most substrate depletion occurs during the hydrolysis-dominant part of the batch, after which additional residence time gives diminishing conversion gains.

Most substrate conversion occurs during the middle portion of the batch, when the policies emphasize hydrolysis-favorable conditions. After this period, substrate depletion slows, and additional residence time gives diminishing gains. This explains why long-batch policies can increase conversion slightly, while shorter, balanced policies retain competitive ethanol productivity. For example, the maximum-conversion policy reaches 0.440 conversion at 91.8h, whereas the balanced-knee policy retains 0.434 conversion at 83.8h. Thus, the balanced-knee policy preserves 98.6% of the maximum conversion while shortening the batch by 8.0h. This comparison shows that extending the batch is beneficial only when the added residence time produces meaningful conversion or ethanol gains, as shown in Figure 9.

Taken together, the state trajectories support the biochemical interpretation of the dynamic policies. Early operation supports growth and enzyme formation, intermediate operation promotes hydrolysis and sugar release, and later operation emphasizes sugar consumption and ethanol production. The trajectories therefore connect the numerical Pareto results with the underlying CBP subprocesses of cellulase activity, substrate solubilization, microbial fermentation, sugar accumulation, product inhibition, and batch-time selection.

### 4.9. Feasibility Threshold Sensitivity and Independent-Seed Repeatability

The feasibility threshold sensitivity analysis quantified how strongly the feasible archive depends on the selected minimum conversion requirement. At $X_{\mathrm{conv},\min}=0.35$, 70,651 policies were feasible, corresponding to 58.9% of the evaluated archive. Increasing the threshold to 0.38 reduced the feasible count to 46,343 policies, while a threshold of 0.40 retained 24,620 policies. At the main threshold of 0.42, only 5017 policies remained feasible, corresponding to 4.18% of the archive. Thus, increasing the threshold from 0.35 to 0.42 reduced the feasible policy count by approximately 92.9%.

Despite this sharp reduction in feasible policy count, the maximum attainable ethanol titer remained $1.265\;g\;L^{-1}$ across the attainable thresholds from 0.35 to 0.42. This indicates that the best ethanol-producing region was still present under the main feasibility definition. However, no policies were feasible at thresholds of 0.44 or 0.55, showing that these conversion requirements fall outside the attainable region for the current reduced-order model, operating bounds, and evaluated archive. The threshold of 0.42 is therefore strict but attainable, whereas 0.44 and 0.55 are too restrictive for the present formulation (Table 13).

Because the Pareto search relies on randomized policy initialization and stochastic refinement, independent-seed repeatability was also evaluated. Three additional reduced-budget runs were performed using the same model, constraints, and operating bounds. The maximum ethanol titer was highly consistent across seeds, with values of 1.2627, 1.2637, and $1.2624\;g\;L^{-1}$. This corresponded to a mean of $1.2629\;g\;L^{-1}$ and a standard deviation of approximately $0.0007\;g\;L^{-1}$. Maximum conversion was similarly stable, ranging from 0.4375 to 0.4386, with a mean of approximately 0.4380 and a standard deviation of approximately 0.0006.

**Figure 10:**
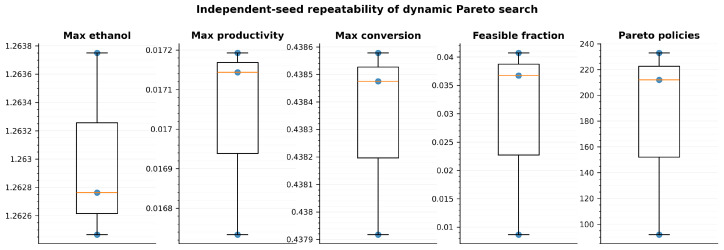
Independent-seed repeatability of the dynamic Pareto search. The repeated runs show that the high-performance region is stable across random initializations, although the number of feasible and non-dominated policies varies with the stochastic search path.

The number of feasible and non-dominated policies varied more strongly across seeds. Feasible policy counts ranged from 165 to 728, while Pareto policy counts ranged from 92 to 221. This indicates that the stochastic search path affects how densely the feasible region is sampled, but not the approximate location of the high-performing ethanol and conversion region. The repeatability results therefore support the conclusion that the main Pareto performance envelope is not an artifact of a single random initialization (Table 14 and Figure 10).

### 4.10. Interpretation of Results and Value for Bioprocess Optimization and Control

The Pareto results reveal coupled trade-offs among key process variables in CBP operation. Ethanol titer, productivity, and substrate conversion are positively correlated over much of the feasible space, but they favor different batch durations and operating policies. High-titer and high-conversion policies generally require longer batches, whereas high-productivity policies favor earlier termination. Similar behavior has been observed in multi-objective optimization of fed-batch and other bioprocess systems, where yield, titer, productivity, operating time, and cost cannot usually be optimized simultaneously because of competing process requirements [10,22]. For bioethanol production, multi-objective formulations have also shown that time-dependent operation can reveal trade-offs that remain hidden in single-objective analysis [27]. In the present CBP model, low-severity operation remains feasible, but careful dynamic temperature and pH scheduling is required to preserve ethanol performance.

The static baseline analysis further shows that constant-temperature and constant-pH policies cannot satisfy the main feasibility threshold in this model. Dynamic operation is therefore important not only for improving ethanol titer and productivity but also for reaching a stricter conversion target. This supports the use of dynamic optimization rather than fixed-condition screening for CBP operation. Similar arguments have been made in fermentation and biorefinery optimization studies, where time-dependent operating policies can reveal performance improvements and feasibility constraints that are overlooked in static or single-objective formulations [27,28]. More broadly, model-driven fermentation studies emphasize that dynamic models can support scale-up, process monitoring, and operational decision-making when used within a suitable optimization or control framework [5].

These results complement recent modeling and machine-learning studies of CBP processes. Previous work has shown that CBP datasets and predictive models can support endpoint-level analysis of ethanol and co-product formation [9,29]. Other CBP modeling studies have emphasized the need for mechanistic understanding of substrate conversion, microbial behavior, and process limitations [15]. The focus here is different: rather than predicting endpoint performance alone, the Pareto framework maps the within-batch operating landscape and links process priorities directly to dynamic temperature and pH schedules.

The feasibility threshold analysis provides an additional layer of decision support. A conversion threshold of 0.42 is strict but attainable, whereas thresholds of 0.44 and 0.55 are not attainable under the present model and operating bounds. This distinction is important because treating an infeasible target as a normal operating requirement can lead to misleading process conclusions. In this sense, the Pareto framework acts not only as an optimizer but also as a diagnostic tool for identifying realistic performance limits and feasible compromise regions. Pareto-based decision support is widely used in multi-objective process optimization because it allows process engineers to select policies according to explicit priorities rather than relying on a single arbitrarily weighted scalar objective [12,13]. Normalized normal constraint and related methods further illustrate how structured Pareto front generation can support decision-making when objectives conflict [14,30].

The results also clarify how the present approach differs from batch-to-batch learning. The objective is not to update operating policies across repeated batches using endpoint data but to characterize the within-batch dynamic operating landscape through Pareto optimization. The resulting fronts, policy maps, and representative trajectories provide interpretable guidance for selecting operating strategies according to priorities such as high ethanol titer, short batch duration, low severity, or balanced operation. This makes the approach complementary to adaptive learning and model predictive control methods: offline Pareto maps can identify promising operating regions, while future online controllers can adjust selected policies as measurements become available during the batch [10,11,31].

### 4.11. Limitations, Validation Scope, and Future Directions

The findings should be interpreted as model-based optimization outcomes rather than experimentally validated operating recipes. The present study is an in silico dynamic optimization and policy-screening study, and no new pilot-scale experiments were conducted. Instead, the model is used as a reduced-order virtual CBP system to compare alternative temperature and pH policies and to identify trade-offs among ethanol titer, productivity, substrate conversion, sugar accumulation, operating severity, control movement, and batch time.

The kinetic model uses lumped substrate, sugar, biomass, enzyme, and ethanol states, and the resulting Pareto policies depend on the assumed parameterization of temperature and pH effects. This reduced-order structure enables a large-scale dynamic policy search, but it does not resolve feedstock heterogeneity, detailed cellulose depolymerization, intracellular metabolism, microbial community dynamics, or competing fermentation products. More detailed CBP models, including genome-scale, cybernetic, and population-balance formulations, could be used in future work to test whether the same Pareto trade-offs persist under higher-fidelity biological representations [6,7,15].

Although direct experimental validation is outside the scope of the present work, the simulated behavior is consistent with several established features of CBP and lignocellulosic ethanol production. First, the model reproduces the expected coupling between hydrolysis and fermentation: soluble sugars accumulate when hydrolysis proceeds faster than fermentation and are depleted when fermentation becomes dominant. Second, the use of separate temperature and pH activity functions reflects the known conflict between conditions that favor enzymatic hydrolysis and those that favor microbial ethanol production. Third, the inclusion of ethanol inhibition is consistent with batch fermentation behavior, where increasing product concentration can reduce effective fermentation performance. Finally, the static baseline comparison supports the practical motivation for dynamic operation by showing that time-varying temperature and pH profiles can reach performance regions that are not reached by constant-condition policies under the same model assumptions. These literature-consistent behaviors provide contextual support for the proposed optimization framework, but they do not replace experimental validation.

The present study is deterministic and does not include full kinetic-parameter uncertainty propagation. Although the conversion-feasibility threshold was varied to test the robustness of the feasible-policy classification, the kinetic constants themselves were kept fixed during the Pareto search. This limits the robustness interpretation of the absolute ethanol titer, productivity, conversion, and feasible policy counts. Parameters such as $V_{h,\max}$, $Y_{P,\max}$, $k_{I}$, $k_{C}$, $K_{X}$, and the temperature–pH activity centers are expected to be especially important because they directly control hydrolysis, fermentation, inhibition, sugar consumption, and biological activity. Future work should therefore combine experimental parameter estimation with local and global sensitivity analyses, uncertainty propagation, and robust or uncertainty-aware Pareto optimization. Robust multi-objective optimal control and dynamic optimization under uncertainty provide useful methodological foundations for this extension [18,19]. Multi-objective optimal experimental design could also be used to select experiments that improve parameter identifiability while testing informative regions of the Pareto front [11].

Future work should validate selected representative Pareto policies experimentally. A practical next step would be to test a small subset of policies, such as the maximum-ethanol, maximum-productivity, minimum-severity, and balanced-knee policies, in controlled batch or pilot-scale CBP experiments. The resulting time-resolved measurements of the substrate, soluble sugars, biomass, enzyme activity, and ethanol could then be used to recalibrate the kinetic model and update the Pareto front under experimentally calibrated conditions.

Literature-derived CBP datasets could also be connected with dynamic optimization by informing parameter priors, feasible operating ranges, co-product constraints, and experimental policy selection. This would be especially useful because recent multi-product CBP modeling has shown that literature-derived data contain ethanol and non-ethanol outputs with uneven support and missing-label structure [9].

Finally, the offline Pareto policies developed here could be incorporated into model predictive control, allowing temperature and pH trajectories to be adjusted online as sugar and ethanol measurements become available during CBP operation [5,10,31].

## 5. Summary and Conclusions

This study formulated consolidated bioprocessing (CBP) as a feasibility-aware multi-objective dynamic optimization problem. Rather than optimizing only the final ethanol titer, the proposed formulation considered several competing objectives, including ethanol titer, volumetric productivity, substrate conversion, soluble sugar accumulation, operating severity, control movement, and batch time. Time-varying temperature and pH profiles, together with the terminal batch time, were optimized to generate feasible Pareto-optimal CBP operating strategies.

The main optimization retained 120,000 dynamic policies for analysis, of which 5017 satisfied the key feasibility requirement and 328 were identified as feasible Pareto-optimal policies. Within the feasible Pareto archive, the best ethanol titer reached $1.265\;g\;L^{-1}$, the best productivity reached $0.017\;g\;L^{-1}\;h^{-1}$, and the highest substrate conversion was 0.440. Compared with the best criterion-specific static constant-operation baselines, the dynamic Pareto policies improved ethanol titer by 10.6%, productivity by 8.3%, and substrate conversion by 14.3%. These results indicate that dynamic scheduling of temperature and pH can provide measurable advantages over constant-operation policies within the assumptions of the proposed model.

The Pareto-optimal results also show that CBP operation involves structured trade-offs rather than a single universal optimum. Policies favoring a high ethanol titer and high substrate conversion generally required longer batch durations, whereas policies favoring high productivity or a shorter batch time accepted lower final ethanol concentrations. The feasibility threshold analysis further showed that the main conversion threshold of $X_{\mathrm{conv},\min}=0.42$ was stringent but attainable under the present model and operating bounds. In contrast, stricter thresholds of 0.44 and 0.55 were not attainable within the evaluated policy archive. Independent-seed repeatability runs confirmed that the high-performing regions of the ethanol titer and substrate conversion were reproducible across stochastic optimization runs.

Overall, this study introduces a computational framework for generating and evaluating interpretable dynamic CBP policies as a decision-support tool for process design. The results should be interpreted as model-based optimization outcomes rather than experimentally validated operating recipes. Future work should therefore focus on experimental testing of selected Pareto policies, recalibration of the kinetic model using time-resolved CBP data, and extension of the framework to robust or uncertainty-aware Pareto optimization. Integration of economic and life-cycle objectives would also help assess whether the predicted operating advantages translate into practical process-level benefits.

## Figures and Tables

**Table 1:** Positioning of the present study relative to published CBP modeling and Pareto-based bioprocess optimization studies.

Study Category	Typical Focus	Representative Published Studies	Distinction of the Present Work
Foundational CBP studies	Establish microbial cellulose utilization and consolidated bioprocessing as a route for lignocellulosic ethanol production.	[1,2]	Formulates CBP operation as a feasibility-aware dynamic Pareto optimization problem rather than a conceptual or process-development analysis.
Experimental and kinetic CBP studies	Examine cellulolytic fermentation, microbial growth, ethanol production, inhibition, and hydrolysis–fermentation behavior.	[3,8]	Uses published kinetic behavior to motivate a reduced-order dynamic model suitable for large-scale temperature–pH policy screening.
Detailed mechanistic CBP models	Describe enzyme coordination, metabolic behavior, or genome-scale characteristics of cellulolytic organisms.	[6,7]	Uses a computationally compact model to construct feasible Pareto fronts rather than resolving detailed intracellular or enzyme allocation mechanisms.
General Pareto and multi-objective optimization methods	Develop or apply non-dominated sorting, normal boundary intersection, normalized normal constraint, or dynamic multi-objective optimal control methods.	[10,12,13,14]	Applies Pareto search to CBP-specific dynamic operating policies with temperature, pH, and batch time as decision variables.
Present study	Feasibility-aware dynamic Pareto optimization of CBP operation.	This work	Generates CBP-specific feasible Pareto fronts, representative temperature–pH policy maps, static baseline comparison, conversion threshold sensitivity, and independent-seed repeatability analysis.

**Table 2:** Dynamic model states and manipulated variables.

Symbol	Description	Role
X(t)	Biomass concentration	State
E(t)	Total enzyme activity	State
B(t)	Insoluble substrate pool	State
C(t)	Soluble sugar pool	State
P(t)	Ethanol concentration	State
T(t)	Reactor temperature	Manipulated variable
pH(t)	Reactor pH	Manipulated variable
$t_{f}$	Final batch time	Decision variable

**Table 3:** Origin and role of the kinetic parameter groups used in the reduced-order CBP model.

Parameter Group	Model Role	Source/Rationale	Representative References
Growth parameters	Biomass growth and carrying capacity	Literature-informed nominal values	[8,16]
Enzyme parameters	Enzyme formation and deactivation	Reported CBP enzyme production behavior	[3,7]
Hydrolysis parameters	Insoluble substrate conversion to soluble sugar	Reduced-order hydrolysis kinetics	[7,8]
Sugar consumption parameters	Sugar depletion during hydrolysis-dominant operation	Nominal CBP mass-balance behavior	[1,8]
Fermentation parameters	Sugar conversion to ethanol	Literature-based fermentation trends	[8,16]
Inhibition parameters	Ethanol-related reduction in process activity	Reported inhibition behavior in fermentation models	[8,16]
Temperature–pH activity parameters	Activity correction for growth, hydrolysis, and fermentation	Nominal operating optima from the CBP literature	[3,7,17]
Phase transition parameters	Smooth transition among batch stages	Chosen to represent staged CBP progression	[5,10]

**Table 4:** Interpretation of the phase-weighting functions in the reduced-order CBP model.

Weight	Dominant Model Role	Biological Interpretation
$\omega_{1}\left(t\right)$	Growth and enzyme formation	Early dominance of biomass growth and cellulase/enzyme production
$\omega_{2}\left(t\right)$	Substrate hydrolysis	Intermediate dominance of insoluble substrate solubilization and sugar release
$\omega_{3}\left(t\right)$	Fermentation	Later dominance of sugar consumption, ethanol formation, and inhibition effects

**Table 5:** Nominal temperature and pH activity parameters used for growth, hydrolysis, and fermentation.

Process	$T_{z}^{*}$	$\sigma_{T,z}$	$\phi_{\min}$	$\phi_{\max}$	${\mathrm{pH}}_{z}^{*}$	$\sigma_{\mathrm{pH},z}$	$\psi_{\min}$	$\psi_{\max}$
Growth/enzyme formation	48.0	12.5	0.15	1.25	5.6	0.85	0.15	1.25
Hydrolysis	50.0	13.0	0.15	1.25	5.2	0.75	0.15	1.25
Fermentation	42.0	11.0	0.12	1.20	6.0	0.90	0.15	1.20

**Table 6:** Nominal kinetic constants and correction factors used in the reduced-order CBP simulations.

Parameter	Symbol	Nominal Value	Description
Maximum growth coefficient	$\mu_{\max}$	$0.215\;h^{-1}$	Base growth coefficient before temperature–pH correction
Maximum enzyme formation coefficient	$Y_{E,\max}$	0.070	Base enzyme formation coefficient before activity and pretreatment correction
Maximum hydrolysis coefficient	$V_{h,\max}$	$0.235\;h^{-1}$	Base hydrolysis coefficient before activity and substrate correction factors
Hydrolysis saturation constant	$K_{h}$	$0.85/(\chi_{\mathrm{pret}}\chi_{S})$	Apparent saturation constant for insoluble substrate hydrolysis
Maximum ethanol production coefficient	$Y_{P,\max}$	$0.215\;h^{-1}$	Base ethanol production coefficient before temperature–pH correction
Ethanol inhibition coefficient	$k_{I}$	$0.045\;L\;g^{-1}$	Inhibition coefficient reducing fermentation rate as ethanol accumulates
Sugar consumption coefficient	$k_{C}$	$0.160\;h^{-1}$	Sugar consumption term during the hydrolysis-dominant phase
Base deactivation coefficient	$k_{d,0}$	$0.009\;h^{-1}$	Base loss term used in the biomass-growth expression
Base enzyme deactivation coefficient	$k_{E,0}$	$0.010\;h^{-1}$	Base enzyme deactivation coefficient before temperature correction
Biomass carrying capacity	$K_{X}$	7.5	Carrying capacity term in the biomass growth equation
Pretreatment correction factor	$\chi_{\mathrm{pret}}$	1.10	Nominal factor representing the pretreated-substrate case
Substrate-loading correction factor	$\chi_{S}$	1.00	Nominal substrate-loading factor used in the main simulations

**Table 7:** Objective functions for the dynamic Pareto optimization problem.

Objective	Mathematical Form	Direction	Interpretation
Ethanol titer	$P(t_{f})$	Maximize	Final ethanol concentration
Productivity	$\left[P\left(t_{f}\right)-P\left(0\right)\right]/t_{f}$	Maximize	Volumetric ethanol productivity
Conversion	$\left[B\left(0\right)-B\left(t_{f}\right)\right]/B\left(0\right)$	Maximize	Fraction of insoluble substrate consumed
Sugar accumulation	$t_{f}^{-1}\int_{0}^{t_{f}}C\left(t\right)\;dt$	Minimize	Average soluble sugar buildup
Severity	$t_{f}^{-1}\int_{0}^{t_{f}}s_{\mathrm{sev}}\left(t\right)\;dt$	Minimize	Temperature and pH operating severity
Control movement	$\sum_{j=1}^{N_{u}-1}\left[{\left(\Delta T_{j}\right)}^{2}+{\left(\Delta {\mathrm{pH}}_{j}\right)}^{2}\right]$	Minimize	Smoothness of the operating policy
Batch time	$t_{f}$	Minimize	Duration of the batch

**Table 8:** Feasibility-aware archive-based dynamic Pareto policy-search procedure.

Step	Procedure
1	Generate an initial archive of random dynamic temperature, pH, and batch-time policies within the admissible bounds.
2	Simulate each policy using the reduced-order CBP dynamic model from t=0 to $t=t_{f}$.
3	Compute ethanol titer, productivity, conversion, sugar accumulation, operating severity, control movement, and batch time.
4	Assign the binary feasibility flag F(π) based on the conversion and sugar constraints.
5	Apply non-dominated sorting only to policies with F(π)=1 using Pareto dominance.
6	Select high-performing archive members from the feasible and non-dominated policy set.
7	Generate offspring policies by perturbing and recombining selected archive members.
8	Repeat simulation, feasibility screening, archive update, and Pareto sorting for all generations.
9	Extract the final feasible non-dominated archive as the reported Pareto set.
10	Select representative Pareto policies for maximum ethanol, maximum productivity, maximum conversion, minimum sugar, minimum severity, minimum batch time, and balanced-knee operation.

**Table 9:** Comparison of the present feasibility-aware archive-based policy search with alternative optimization and control frameworks.

Framework	Main Role	Strengths	Limitations for the Present CBP Study
Feasibility-aware archive-based direct policy search	Offline generation of dynamic temperature–pH policies and feasible Pareto fronts	Simple policy encoding, direct simulation of complete batch policies, explicit feasibility screening, retains infeasible policies for diagnostics	Not a standard evolutionary optimizer; convergence is assessed empirically through archive behavior and repeatability
NSGA-II/evolutionary Pareto optimization	Population-based multi-objective optimization using non-dominated sorting and diversity preservation	Well-established Pareto front generation and diversity control [12]	A fair benchmark would require implementing the same policy encoding, feasibility rules, search budget, and objectives
MPC	Online receding-horizon control with feedback from process measurements	Can update temperature and pH trajectories during the batch as measurements become available	Requires calibrated dynamics, online state estimation, and reliable measurements; not available in the present in silico screening study
Reinforcement learning	Data-driven or simulation-trained adaptive policy learning	Can potentially learn nonlinear control policies without explicit scalarization	Requires many training episodes, reward design, safety checks, and a validated simulation or experimental environment
Static constant-operation baseline	Simple benchmark using constant temperature, constant pH, and batch time	Transparent, reproducible, and directly tests whether dynamic operation provides an advantage	Does not adapt temperature or pH over time and may fail feasibility constraints

**Table 10:** Comparison between dynamic Pareto policies and criterion-specific best static constant-operation baselines. The static baselines were selected independently for each performance criterion from the constant-temperature, constant-pH, and batch-time grid. None of the selected static baselines satisfy the main conversion feasibility threshold.

Criterion	Dynamic	Static	Improvement	$T_{\mathrm{static}}$	${\mathrm{pH}}_{\mathrm{static}}$	$t_{f}$
	Value	Baseline	(%)	(°C)	(-)	(h)
Max ethanol titer	1.265	1.144	10.6	48.0	5.5	144.0
Max productivity	0.0170	0.0157	8.3	48.0	5.5	63.0
Max conversion	0.440	0.384	14.3	49.0	5.4	144.0

**Table 11:** Objective-space summary of the feasible Pareto-optimal CBP policies at $X_{\mathrm{conv}}\geq 0.42$. For ethanol titer, productivity, and conversion, higher values are preferred; for average sugar, operating severity, control movement, and batch time, lower values are preferred.

Objective	Best Pareto Value	Median Pareto Value	Worst Pareto Value
Ethanol titer (g L^−1^)	1.265	1.203	0.517
Productivity (g L^−1^ h^−1^)	0.01696	0.01418	0.00838
Substrate conversion (-)	0.4395	0.428	0.420
Average sugar (g L^−1^)	0.236	0.427	0.578
Operating severity (-)	0.027	0.052	0.140
Control movement (-)	0.0038	0.0230	0.1436
Batch time (h)	51.5	80.4	143.9

**Table 12:** Representative feasible Pareto-optimal dynamic CBP policies.

Policy	Ethanol	Prod.	Conv.	Avg. Sugar	Severity	Movement	Time
	(g L^−1^)	(g L^−1^ h^−1^)	(-)	(g L^−1^)	(-)	(-)	(h)
Max ethanol	1.265	0.0124	0.427	0.353	0.044	0.022	101.7
Max productivity	1.158	0.0170	0.430	0.473	0.054	0.030	68.3
Max conversion	1.214	0.0132	0.440	0.431	0.076	0.047	91.8
Min sugar	1.206	0.0084	0.420	0.236	0.052	0.030	143.9
Min severity	1.255	0.0121	0.420	0.325	0.027	0.016	104.0
Min batch time	0.518	0.0101	0.427	0.544	0.075	0.015	51.5
Balanced knee	1.216	0.0145	0.434	0.416	0.046	0.023	83.8

**Table 13:** Sensitivity of feasible policy counts to the minimum conversion threshold.

Min. Conversion	Feasible Policies	Feasible Fraction	Max Ethanol	Max Conversion
Threshold			(g L^−1^)	(-)
0.35	70,651	0.589	1.265	0.4395
0.38	46,343	0.386	1.265	0.4395
0.40	24,620	0.205	1.265	0.4395
0.42	5017	0.0418	1.265	0.4395
0.44	0	0.000	–	–
0.55	0	0.000	–	–

**Table 14:** Independent-seed repeatability of the dynamic Pareto optimization.

Seed	Evaluated Policies	Feasible Policies	Pareto Policies	Max Ethanol	Max Conversion
				(g L^−1^)	(-)
1042	19,000	698	212	1.2627	0.4386
2042	19,000	165	92	1.2637	0.4379
3042	19,000	728	221	1.2624	0.4375

## Data Availability

The original contributions presented in this study are included in the article. Further inquiries can be directed to the corresponding author.
